# Cell carbon content and biomass assessments of dinoflagellates and diatoms in the oceanic ecosystem of the Southern Gulf of Mexico

**DOI:** 10.1371/journal.pone.0247071

**Published:** 2021-02-17

**Authors:** Lorena Linacre, Citlalli Sánchez-Robles, Uriel Mirabal-Gómez, J. Rubén Lara-Lara, Carmen Bazán-Guzmán

**Affiliations:** Departamento de Oceanografía Biológica, División de Oceanología, Centro de Investigación Científica y de Educación Superior de Ensenada, Baja California (CICESE), Ensenada, Baja California, México; Stazione Zoologica Anton Dohrn, ITALY

## Abstract

This study assessed the cell carbon content and biomass for genera of dinoflagellates and diatoms in the oceanic ecosystem of the Southern Gulf of Mexico. Carbon content estimates were based on biovolume calculations derived from linear dimension measurements of individual cells and the approximate geometric body shape of each genus. Then, biomass assessments were performed for both groups in two gulf regions (Perdido and Coatzacoalcos) using these carbon content factors and cell abundances. After four seasonal cruises, 11,817 cells of dinoflagellates and 3,412 cells of diatoms were analyzed. Diverse body shapes and cell sizes were observed among 46 dinoflagellate genera and 37 diatom genera. Nano-cells of dinoflagellates (68% <20 μm) and micro-cells of diatoms (77% 20–200 μm, mostly 50–75 μm) were predominant. According to this cell-size structure, on average, diatoms contained 40% more carbon per cell than dinoflagellates. Contrasting carbon content estimates were observed within the genera of both microalgae. Large carbon averages (>10,000 pg C cell^-1^) were attributed to Gonyaulacal and some occasional genera of dinoflagellates (e.g., *Pyrocystis* and *Noctiluca*) and centric diatoms. In contrast, values up to 3 orders of magnitude lower were found for Peridinial and Gymnodinial dinoflagellates and pennate diatoms. Based on these carbon content estimates, which can be considered representative for most of this oceanic ecosystem, seasonal and regional differences were found in the biomass assessments conducted for these functional groups. Overall, dinoflagellates (mostly low-carbon Gymnodinales) had larger depth-integrated biomass than diatoms (mainly rich-carbon centric forms) within the euphotic zone. An exception to it was the late-summer cruise at the Coatzacoalcos region when a surface bloom of centric diatoms was observed in stations influenced by river runoff. This work contributes useful reference information for future ecological studies and models for understanding the biogeochemical functioning of this open-ocean ecosystem.

## Introduction

Carbon is the main structural element of all living organisms that flows through the food webs in marine ecosystems. Hence, it is a useful parameter to understand the role of key functional groups within the plankton community and represent them in marine ecosystem models [1]. For that purpose, relative biomass assessments in terms of carbon are needed. However, calculation of cell carbon content based on the biovolume of each plankton group [2] is challenging due to large variation in cell size across populations. In particular, the phytoplankton community is composed of diverse taxa containing a wide range of size classes (mostly from pico- to micro-cell-sized). Groups such as dinoflagellates and diatoms vary broadly in body shape, thus adding complexity to the calculation of cell biovolume to derive an appropriate carbon factor.

The Southern Gulf of Mexico (SGoM) ecosystem harbors a large diversity of dinoflagellate and diatom species. All the knowledge about the taxonomic composition of these phytoplankton groups, including extensive checklists of species, derives from many years of research [3–18]. However, these studies were conducted mostly in coastal areas and are limited in their scope and conclusions about biogeochemical processes, mainly due to the lack of carbon estimates for microalgae. These data could be missing because of the time-consuming effort involved in measuring individual cells under the microscope for calculating biovolume based on their closest geometric shapes [19–21], the necessity of powerful microscopes to have more accurate measurements of cell lineal dimensions, the skills of the analyst, and the scarcity of precise cell counts in oligotrophic areas where phytoplankton abundance is low [22]. The oceanic waters of SGoM—a region characterized by limited nutrients, low chlorophyll concentration, and relatively isolated from coastal eutrophic waters [23, 24]—are no exception of this lack of information. Therefore, the main objective of the present work is to assess the per-cell carbon content for several genera of diatoms and dinoflagellates commonly recorded within the oceanic ecosystem in SGoM waters, and then evaluate the carbon biomass contribution of both groups in two open-ocean regions (Perdido and Coatzacoalcos) over three seasonal cruises. This effort certainly represents a major first step for future plankton modeling studies and biogeochemical carbon budgets in this oligotrophic marine ecosystem.

## Material and methods

### Phytoplankton sampling

**Fig 1:**
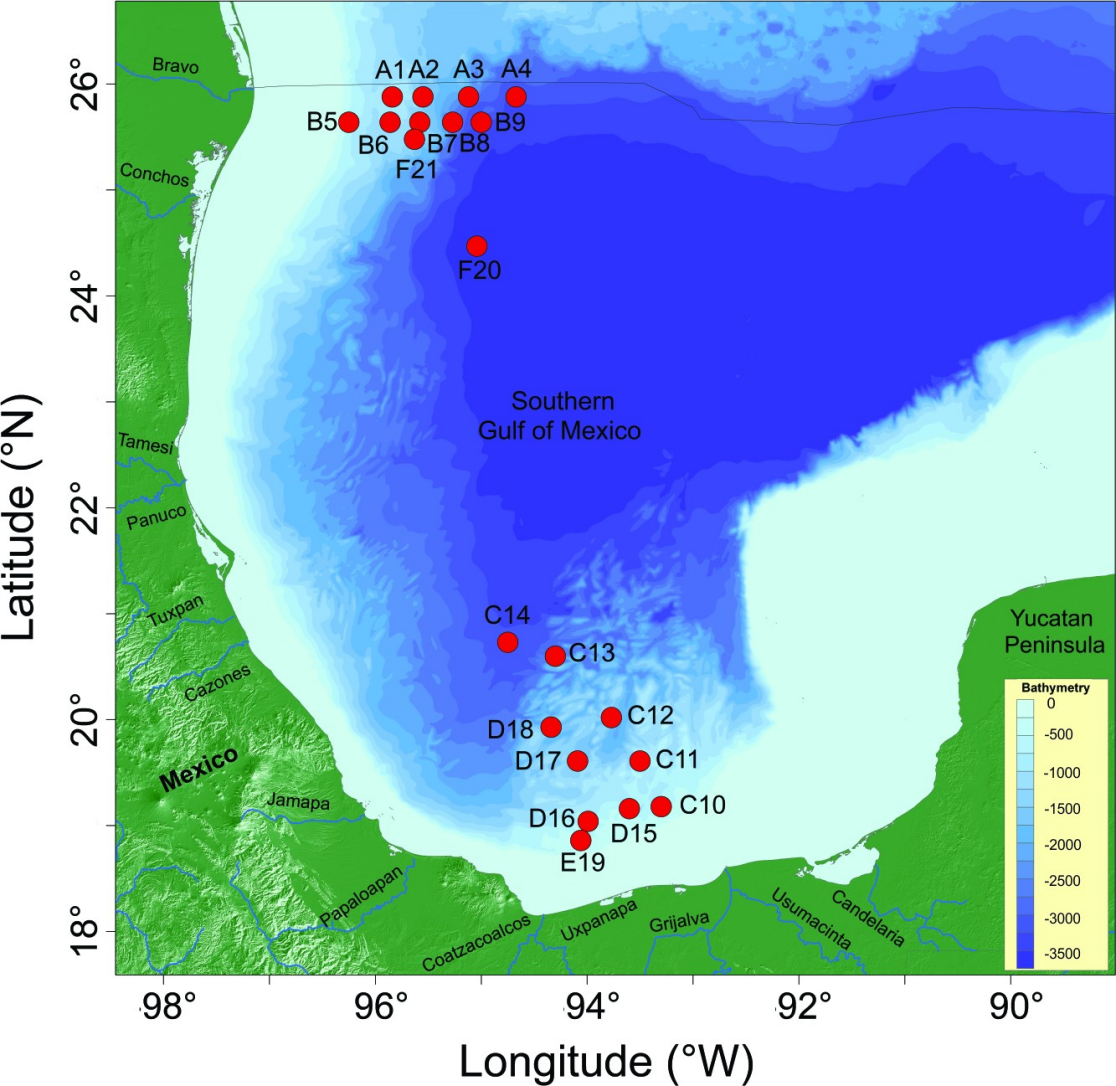
Study area in the Southern Gulf of Mexico (SGoM). Location of the study regions (Perdido and Coatzacoalcos) in oceanic waters over the continental slope (>500 m isobaths) and the deep region (>1,000 m) of the Southern Gulf of Mexico (SGoM). The nominal stations for the phytoplankton sampling conducted during "*Malla Fina*" (MF) cruises are shown for both sites.

Four oceanographic campaigns called "*Malla Fina*" (MF) were conducted in two oceanic regions of the SGoM: Perdido (~25°53’ N to 25°38’ N and 94°40’W to 96°15’ W and also station F20 at 24°28’ N, 95°02’W) and Coatzacoalcos (~18°52’ N to 20°44’ N and 93°18’W to 94°45’ W; Fig 1), during March 2016 (MF1 cruise, late winter), September–October 2016 (MF2 cruise, late summer), May–June 2018 (MF3 cruise, spring) and May 2019 (MF4 cruise, spring). CTD/rosette casts and water sampling were conducted at 17, 19, 18, and 5 stations located beyond the 500 m isobath during MF1, MF2, MF3 and MF4, respectively (Fig 1). Continuous profiles of conductivity, temperature, pressure, dissolved oxygen, chlorophyll fluorescence, and photosynthetically active radiation (PAR, 400–700 nm) were recorded at each station. One-liter seawater samples were collected in amber bottles from 8 depth levels within the euphotic zone (up to 0.1% surface irradiance) for phytoplankton enumeration, taxa identification, and cell measurements. Besides, vertical net-tow samples for supplementary taxonomic analysis were collected using a 20 μm-mesh plankton net deployed from 150 m depth to the surface. In the field, phytoplankton water and net samples were preserved with 4 mL acid Lugol and 4 mL neutralized formaldehyde (1% final concentration), respectively [25], then labeled and stored until analysis at the laboratory.

### Phytoplankton water sample preparation

The 1 L phytoplankton samples were concentrated to obtain a significant number of organisms (≥300 cells). The concentration process was performed using a technique implemented in the Microscopy and Cytometry Laboratory at CICESE (Centro de Investigación Científica y Educación Superior de Ensenada, BC). Briefly, Lugol-fixed samples were poured into 1 L graduated cylinders capped with a plastic seal to avoid evaporation and left to stand during 3 to 4 days to concentrate the cells at the bottom by sedimentation. Then, using a filtration system consisting of a peristaltic pump and silicone tubing with a filtering support at one end, the supernatant liquid was carefully removed by filtration at low suction pressure through a 5 μm-membrane filter to avoid loss of phytoplankton cells until a 100 mL concentrate was reached. Additionally to acid Lugol, 1 mL of neutralized formaldehyde (1% final concentration) was added to the concentrated sample to avoid cell destruction during the taxonomic analysis. A 20-to-50 mL aliquot was then left to settle in chambers for 24 hours and then examined under an inverted microscope using the Utermöhl method [26]. To estimate abundances, the quantified cells were standardized for sample volume following the equation: *Cells L*^*-1*^ = N * (A_*t*_ */ A*_*c*_) * (1000 / (V * d)); where *N* is the number of cells of a specific genus counted, *A*_*t*_ is the total area of the counting chamber (mm^2^), *A*_*c*_ is the counted area of the counting chamber (mm^2^), *V* is the counting chamber volume (mL), and *d* is the sample concentration factor [27].

### Phytoplankton imaging and cell measurements

Phytoplankton samples were examined under a Nikon Eclipse-Ti inverted microscope equipped with a fully motorized stage controlled by the specialized NIS Element AR software (Nikon). Using the Acquisition and Analysis Modules within the NIS Element software, digital images were captured with a monochrome (DS-Qi2) digital camera at 200×, 400×, and 600× magnifications using automated image acquisition. One-hundred random visual fields on each sample were captured using a Z-axis depth control system (Perfect Focus System, PFS) to produce a single completely focused image. Then, each cell in each image was outlined, counted, and sized (either length [L] or height [H], and width [W]) using automated acquisition of morphometric data. The main genera of diatoms and dinoflagellates observed in each image were identified using the taxonomic guides of [3, 17, 28, 29] and the species checklists for Gulf of Mexico waters reported by [10, 11, 13, 30, 31]. Dinoflagellates were classified to order according to the Algaebase webpage latest update [32]. Diatoms were classified according to structural shape as "centric" and "pennate" [29].

### Biovolume, carbon content, and biomass calculations

From the linear dimensions measured in diatom and dinoflagellate cells (i.e., L or H, and W; μm), cell biovolume (BV, μm^3^) was calculated using the appropriate geometric formula according to the body shape of each genus, using the equations proposed by [19–21, 33]. The third dimension, cell "thickness" (also called the "hidden dimension" HD), was measured in a few cases from a subset of cells or was roughly estimated in some genera as a factor from a known linear measurement, following some approximations performed in the literature [20, 22]. Some genera showed more complicated geometric shapes, hence BV was computed as the sum of the partial geometric bodies [21]. All geometric shapes, formulas, and HD approximations used in this work are indicated in S1 Table. The few basic geometric shapes used here do not allow classifying the large variability of phytoplankton body shapes; however, the goal is to find those that reflect, as far as possible, each shape of microalgae with few linear measurements. Once cell BV was calculated, carbon content was assessed using conversion factors from the literature for diatoms only (pg C cell^-1^ = 0.288 * BV^0.811^) and non-diatom protists that include dinoflagellates (pg C cell^-1^ = 0.216 * BV^0.939^) [2]. These comprehensive relationships cover a broad range of cell sizes and predict carbon content based on cell biovolume of live and fixed cells. Finally, cell abundance (cells L^-1^) of diatom and dinoflagellate genera per cruise was converted to biomass (μg C L^-1^) using the carbon content factors (pg C cell^-1^ * 10^−6^ = μg C cell^-1^) estimated for both groups.

### Data analysis

For comparisons between "genera" belonging to dinoflagellate orders and diatom shapes, all linear measurements (μm), BV (μm^3^), and carbon per cell (pg C cell^-1^) from the four seasonal cruises conducted in the Perdido and Coatzacoalcos regions (SGoM open waters), were grouped, log-transformed (Log_10_), and averaged. The greatest linear dimension (GLD, μm) of dinoflagellates and diatoms was binned into ten and fifteen size classes, respectively, which comprised the size categories of nano- (<10–20 μm), micro- (20–200 μm), and meso-cells (>200 μm). Carbon content values (pg C cell^-1^) were binned into five classes in both cases. Frequency distribution histograms were constructed for the whole data set of body GLD and carbon content. Log-transformed averages (standard deviation, ±SD) of carbon content per cell were derived from individual BV computed from the multiple organisms measured (i.e., more than 30 cells per genus). For occasional genera (i.e., <30 cells), only minimum and maximum values were considered to display carbon-per-cell estimates.

In order to evaluate the seasonal carbon contribution of dinoflagellate orders and diatom shapes, pie diagrams per cruise were generated with the median values of integrated carbon biomass (mg C m^-2^) in the euphotic zone (surface to ~150 m depth). Besides, box-plots per cruise and region of depth-integrated carbon biomass (mg C m^-2^) were built for both groups, as well as for the most representative dinoflagellate orders and centric and pennate diatoms. For temporal comparison purposes with a similar sample resolution, only data from MF1 (late winter 2016), MF2 (late summer 2016), and MF3 (spring 2018) cruises were included. Biomass values (μg C L^-1^) from the euphotic zone were depth-integrated using the Trapezoidal Rule. The temporal and spatial variability of hydrographic conditions in the upper euphotic zone was characterized through box plots of temperature (°C) and salinity per cruise using data recorded at 11–17 m depth (~50% surface irradiance).

## Results and discussion

### Basin estimates of cell carbon content for dinoflagellates and diatoms

During the four seasonal cruises, a total of 11,817 and 3,412 cells of dinoflagellates and diatoms, respectively, were measured in linear dimensions for cell-size, BV and carbon-content determinations. Organisms were classified taxonomically into 46 genera of dinoflagellates (belonging to 10 orders) and 37 genera of diatoms (19 centric and 18 pennate) (Table 1). Given the spatial and temporal span of our observations, our cell-size measurements involve regional and seasonal variability, making BV and carbon estimates more comprehensive and representative for most oceanic waters in the SGoM ecosystem. Regional variability arises from two oceanic zones influenced by mesoscale activity, which regulates the thermohaline properties of the upper layer. The remnant structures of anticyclonic eddies that episodically detach from the Loop Current and propagate westward influence the hydrographic conditions of the Perdido region [34]. In the Campeche area, the local dynamics is driven by a semi-permanent cyclonic eddy in the Coatzacoalcos region [35]. Seasonal variability arises from the unique hydrography of the water column at the times of sampling. Spring-summer stratification and winter mixing convection modulate the mixed-layer depth and, consequently, the vertical distribution of the chemical and biological properties [24, 36]. Additionally, meteorological and hydrological events such as intense northerly winds in wintertime (e.g., during MF1), and the large freshwater runoff from the Coatzacoalcos river and Grijalva-Usumacinta system during the rainy season (summer-autumn) (e.g., MF2 at Coatzacoalcos), or from the Mississippi river in spring (e.g., MF4 at Perdido) [36–39], are also sources of temporal variability in our morphometric information. All these processes characterize the environmental conditions reflected in our data over the southern basin. Thus, spatio-temporal and life-cycle variations of the phytoplankton cells embedded in the basin variability may lead to inaccurate average BV computations [19]. Nonetheless, we consider that the sampling during different periods of the year and in various regions and depths of the SGoM brings the opportunity to estimate a more significant number of cells per genus and get more robust assessment of carbon averages for these two microalgae groups, which can be used as a baseline for the whole oceanic GoM ecosystem.

**Table 1:** Genera of dinoflagellates and diatoms identified and measured in lineal dimensions.

Dinoflagellates	Number of organisms measured	Diatoms	Number of organisms measured
**Amphidiniales**		**Centric**	
*Amphidinium*	209	*Asterolampra*	57
**Dinophysales**		*Asteromphalus*	61
*Amphisolenia*	11	*Bacteriastrum*	14
*Dinophysis*	141	*Cerataulina*	19
*Histioneis*	26	*Chaetoceros*	154
*Ornithocercus*	64	*Coscinodiscus*	35
*Oxyphysis*	14	*Dactylosolen*	141
*Phalacroma*	12	*Dytilum*	1
**Gonyaulacales**		*Eucampia*	146
*Alexandrium*	509	*Gossleriella*	3
*Centrodiniuim*	6	*Guinardia*	75
*Ceratocorys*	42	*Hemiaulus*	208
*Cladopyxis*	23	*Leptocylindrus*	28
*Gonyaulax*	191	*Odontella*	2
*Lingulodinium*	69	*Planktoniella*	41
*Protoceratium*	1	*Proboscia*	27
*Pyrophacus*	39	*Rhizosolenia*	110
*Tripos*	136	*Skeletonema*	52
**Gymnodiniales**		*Thalassiosira*	280
*Achradina*	19	**Pennate**	
*Akashiwo*	10	*Cylindrotheca*	32
*Asterodinium*	2	*Diploneis*	12
*Brachidinium*	1	*Entomoneis*	23
*Ceratoperidinium*	1	*Fragilaria*	6
*Cochlodinium*	13	*Grammatophora*	1
*Gymnodinium*	3,243	*Gyrosigma*	1
*Gyrodinium*	1,099	*Haslea*	182
*Karenia*	337	*Lioloma*	5
*Karlodinium*	150	*Mastogloia*	80
*Lepidodinium*	714	*Meuneria*	48
*Nematodinium*	153	*Navicula*	314
*Polykrikos*	4	*Nitzschia*	584
*Torodinium*	321	*Pinnularia*	10
*Warnowia*	15	*Pleurosigma*	26
**Noctilucales**		*Pseudo-nitzschia*	314
*Kofoidinium*	5	*Thalassionema*	175
*Noctiluca*	2	*Thalassiotrix*	2
*Pronoctiluca*	56	*Tropidoneis*	4
**Peridiniales**			
*Corythodinium*	6		
*Heterocapsa*	664		
*Oxytoxum*	919		
*Peridinium*	1		
*Podolampas*	121		
*Protoperidinium*	163		
**Prorocentrales**			
*Prorocentrum*	109		
**Pyrocystales**			
*Pyrocystis*	17		
**Thoracosphaerales**			
*Goniodoma*	2		
*Scrippssiella*	210		
*Pentapharsodinium*	21		
**Tovelliales**			
*Katodinium*	228		

Phytoplankton samples were collected from two oceanic regions (Perdido and Coatzacoalcos) of the SGoM during four cruises conducted in late winter (MF1), late summer (MF2), and spring (MF3 and MF4).

### Size structure and diversity of body shapes

**Fig 2:**
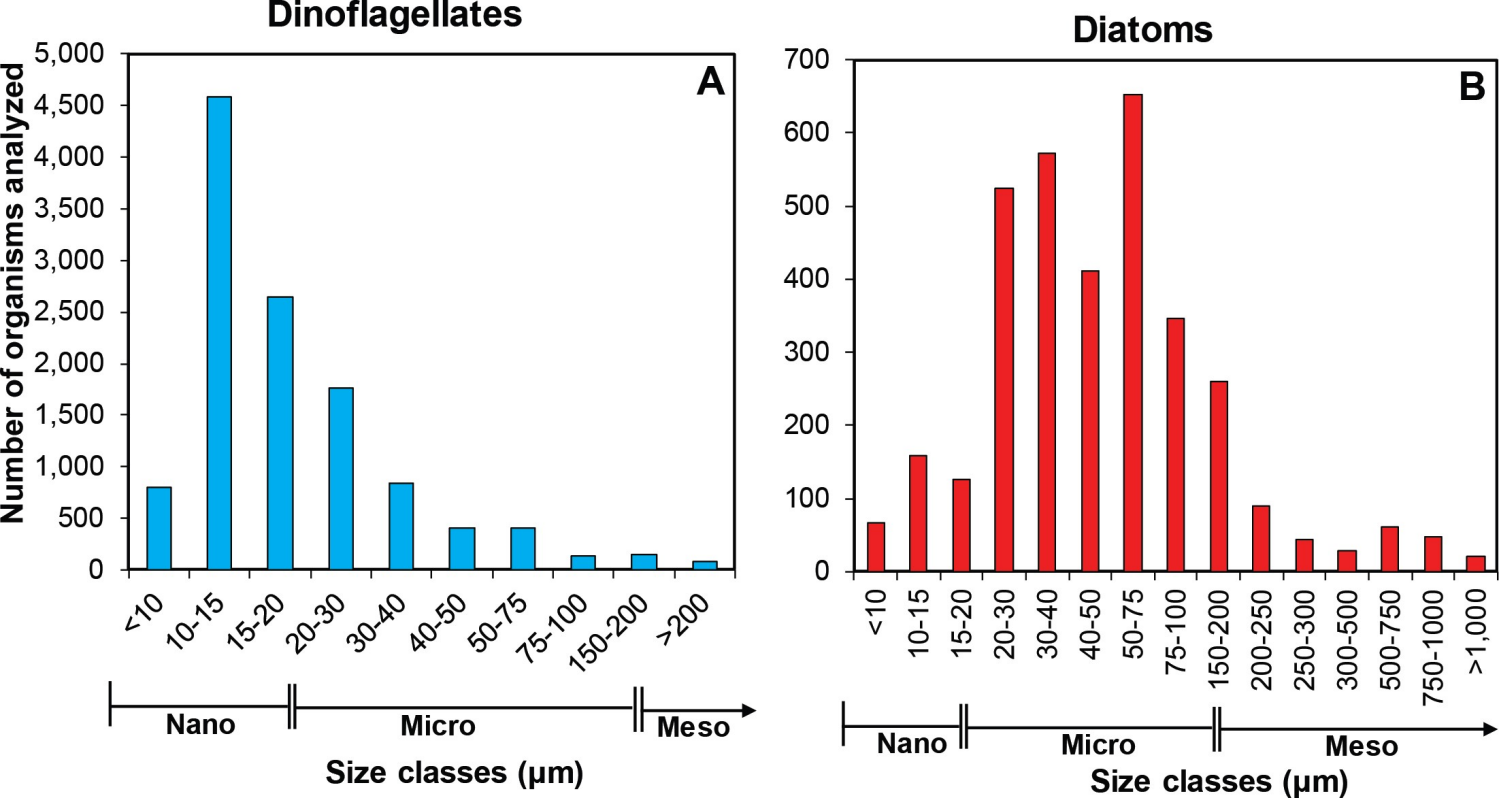
Distribution of the greatest linear dimension (GLD, μm) of two phytoplankton groups. Frequency histograms of cell size indicated by the GLD (μm) measured in genera of (A) dinoflagellates and (B) diatoms. Nano-, micro-, and meso-size categories are displayed at the X-axis. Note the larger bin numbers used for the meso-size class in diatoms.

Due to the variety of body shapes among dinoflagellates and diatoms, broad variations in cell size are found in SGoM natural assemblages. Except for some large-sized genera (GLD >1,000 μm) such as *Amphisolenia* and *Pyrocystis*, dinoflagellates were generally smaller than diatoms (average cell size of 18 and 50 μm, respectively). Most dinoflagellates (68%) comprised nano-cell sizes (<20 μm) and were represented mainly by Gymnodinial genera (e.g., *Gymnodinium*, *Karlodinium*) (Fig 2A). A substantial fraction of diatoms (77%) fell into micro-cell sizes (20–200 μm), being 50–75 μm the most frequent size-range. A few genera of diatoms (e.g., *Rhizosolenia*) also spanned into the meso-size category (Fig 2B). The size structure of the phytoplankton community responds to environmental (light, nutrient availability, vertical mixing) and biotic forcings (differential growth rates, competitive interactions, grazing impact, cell sinking), and hence, strongly influences the function of aquatic ecosystems [40, 41]. Thus, the cell-size distribution found in this study, shaped mainly by the oligotrophy of the oceanic SGoM waters [24], evidences the predominance of small diatom and dinoflagellate components structuring the microbial food webs in this oceanic ecosystem, through which carbon is cycled within the upper layers.

**Fig 3:**
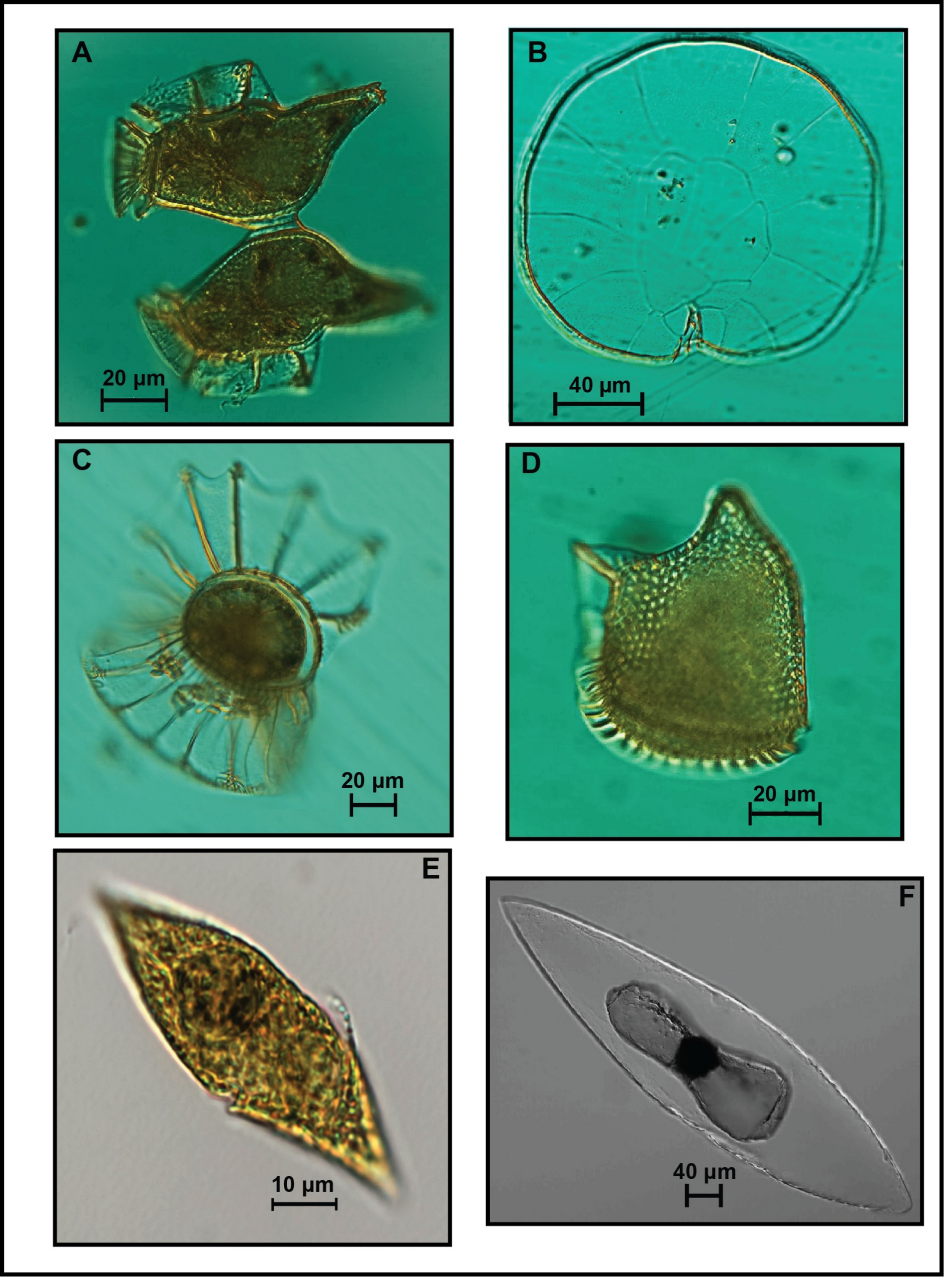
Microscope images of dinoflagellate genera collected in oceanic waters of the SGoM. (A) *Dinophysis*, (B) *Pyrophacus*, (C) *Ornithocercus*, (D) *Phalacroma*, (E) *Gyrodinium*, and (F) *Pyrocystis*. Scale bars are indicated for each picture.

**Fig 4:**
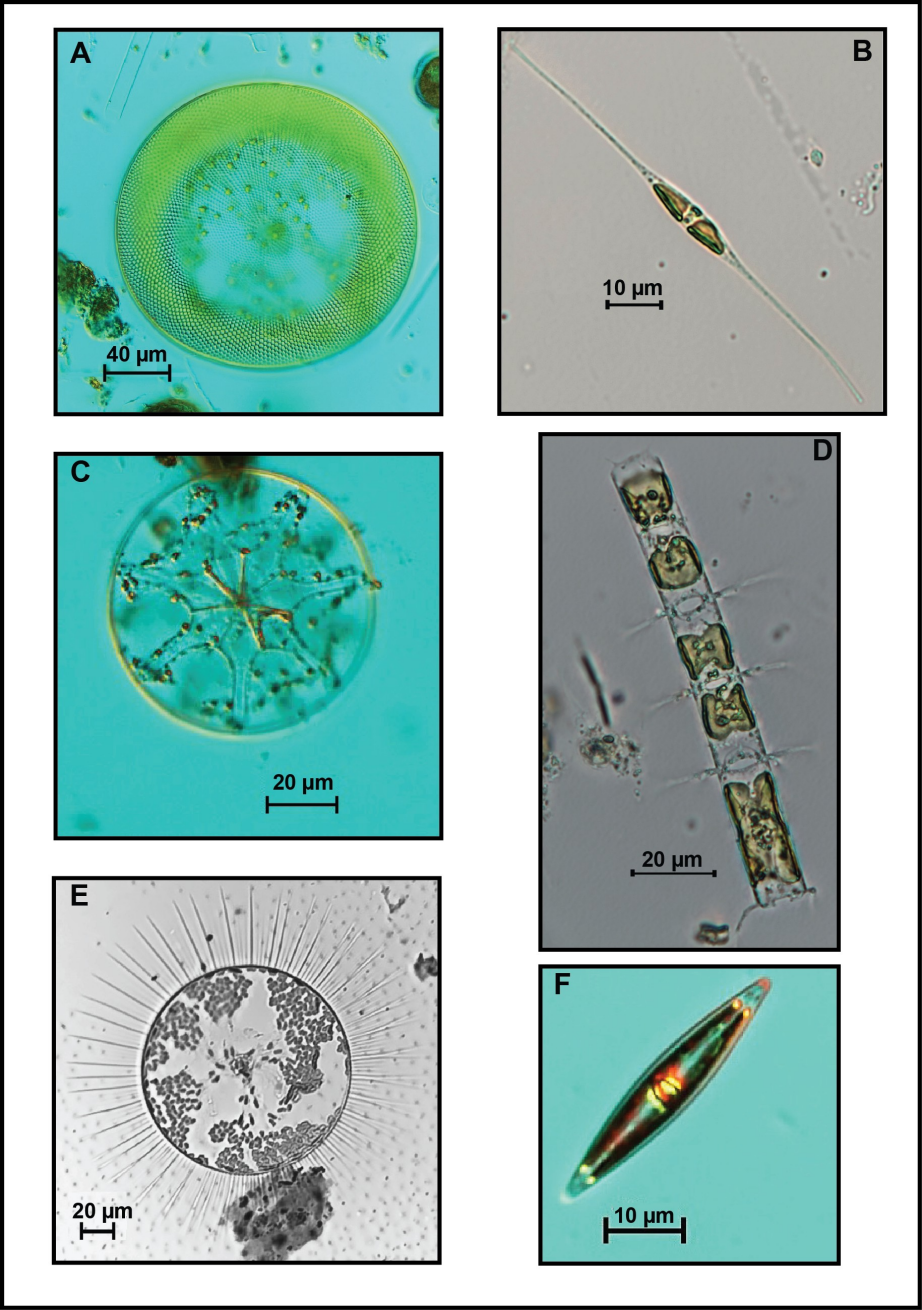
Microscope images of diatom genera collected in oceanic waters of the SGoM. (A) *Coscinodiscus*, (B) *Cylindrotheca*, (C) *Asterolampra*, (D) *Chaetoceros*, (E) *Gossleriella tropica*, and (F) *Nitzschia*. Scale bars are indicated for each picture.

Sample images of the diversity of cell sizes and body shapes of the dinoflagellate and diatom genera found in the SGoM are shown in Figs 3 and 4. An ellipsoid body shape is associated with *Pyrocystis* cells (Fig 3F, S1 Table). Species of this large tropical and oceanic organism have been occasionally reported in low abundance in gulf waters [18, 31, 42]. In contrast, a frequent and abundant genus in this ecosystem is *Gyrodinium* [10, 18]. The cells of this naked dinoflagellate measured in the current study were relatively small (~20–30 μm length) and had an approximate double-cone shape (Fig 3E, S1 Table). Moreover, contrasting dimensions and body shapes can also be observed in several diatom genera. Such is the case of *Gossleriella tropica*, a large species (>100 μm diameter) with a cylinder-shaped body (Fig 4E, S1 Table), which has been described as part of the phytoplankton assemblages in open gulf waters [42].

### Structure of cell-carbon factors

**Fig 5:**
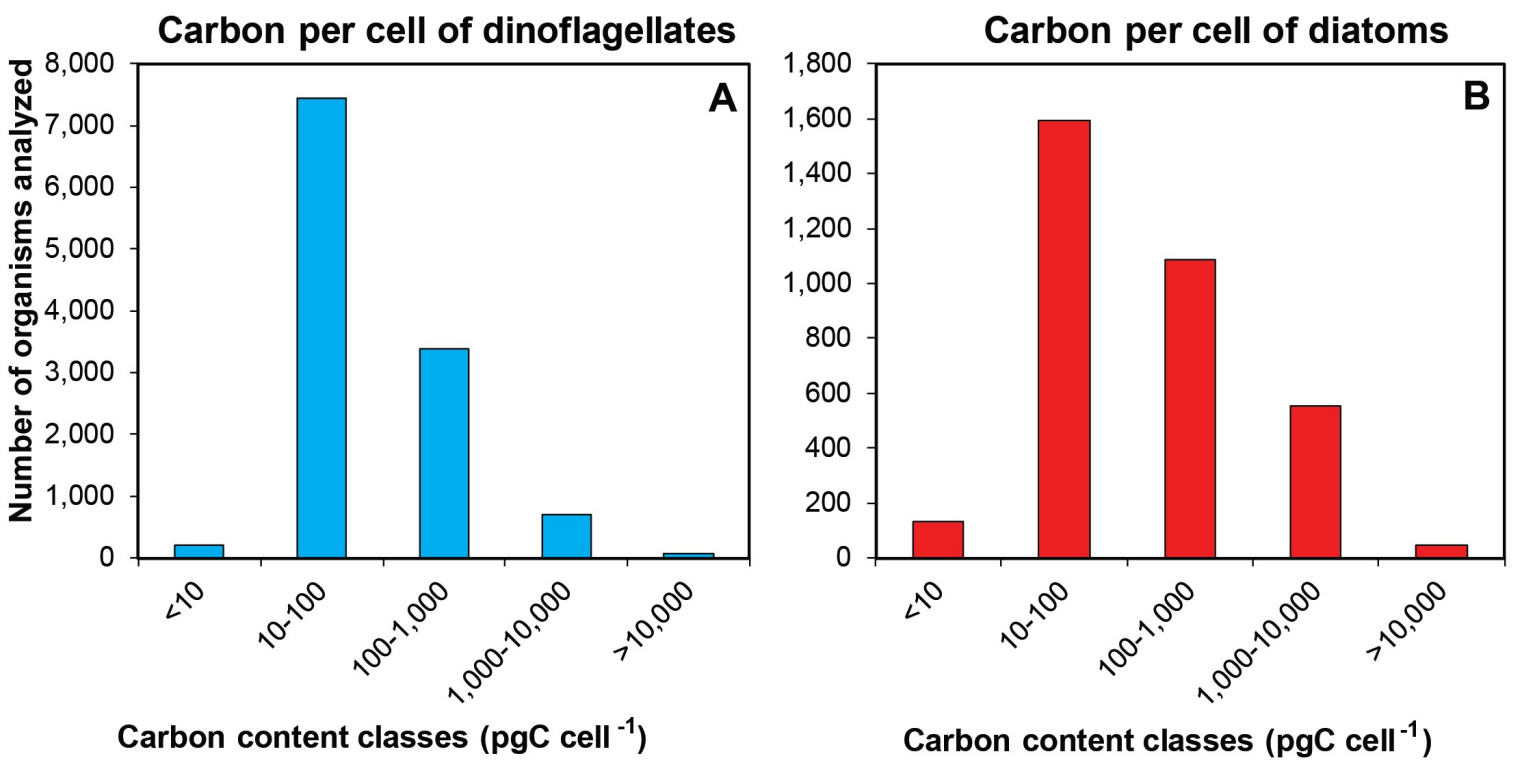
Distribution of carbon content per cell (pg C cell^-1^) of two phytoplankton groups. Frequency histograms of carbon content per cell (pg C cell^-1^) estimated from cell biovolume calculations for genera of (A) dinoflagellates (blue bars) and (B) diatoms (red bars). Five carbon classes are indicated at the X-axis of both groups.

Overall, diatoms have larger cell size, BV, and cell carbon content than dinoflagellates (Figs 2 and 5). The BV values calculated with the whole data set reflect the small size of phytoplankton in this ecosystem. About 72% and 60% of dinoflagellates and diatoms have BV <1,000 μm^3^ and <2,500 μm^3^, respectively. Besides, the predominant shapes in diatoms (e.g., cylinder in centric cells), in contrast to those in dinoflagellates (e.g., flattened ellipsoid or prolate spheroid) (S1 Table), traslate into larger body volumes. Despite the broad spectrum of carbon content in both groups (from 2 up to 10^6^ pg C cell^-1^), dinoflagellates showed less variability in the distribution of carbon content values. Thus, 63% of dinoflagellates fell into the 10–100 pg C cell^-1^ carbon category (Fig 5A). In contrast, most diatoms were divided into two carbon-content classes: 10–100 and 100–1,000 pg C cell^-1^ (47 and 32%, respectively) due to the heterogeneity in cell size (Figs 2B and 5B).

### Specific carbon values for dinoflagellate and diatom genera

The considerable variation in the whole carbon content dataset of each phytoplankton group warrant the need to estimate more specific values, at least at the genus level. Thus, based on the assumption of similar geometric shapes within each genus [19, 21, 33, 43], we classified the carbon content information according to "genus" for dinoflagellates and diatoms. The total cell carbon for each dinoflagellate order and diatom shape included the carbon content of some unidentified genera. However, we first dealt with the broad differences between minimum and maximum carbon values and the positively skewed distribution of the data for the genera of dinoflagellates and diatoms, which added complexity to the estimation of averages and the comparison between them. The logarithmic transformation (Log_10_) as a previous step for average carbon computations (± standard deviation, SD) significantly improved our data toward a normal distribution [2, 44]. Despite this transformation, not all genera of both groups were normally distributed (Shapiro-Wilk test, p<0.05). However, given the large number of cell measurements in this study, especially for the common dinoflagellates and diatoms in the SGoM ecosystem, the log_10_-transformed data tended to be more homogeneously distributed, thus allowing better comparisons among the different genera in both groups.

**Fig 6:**
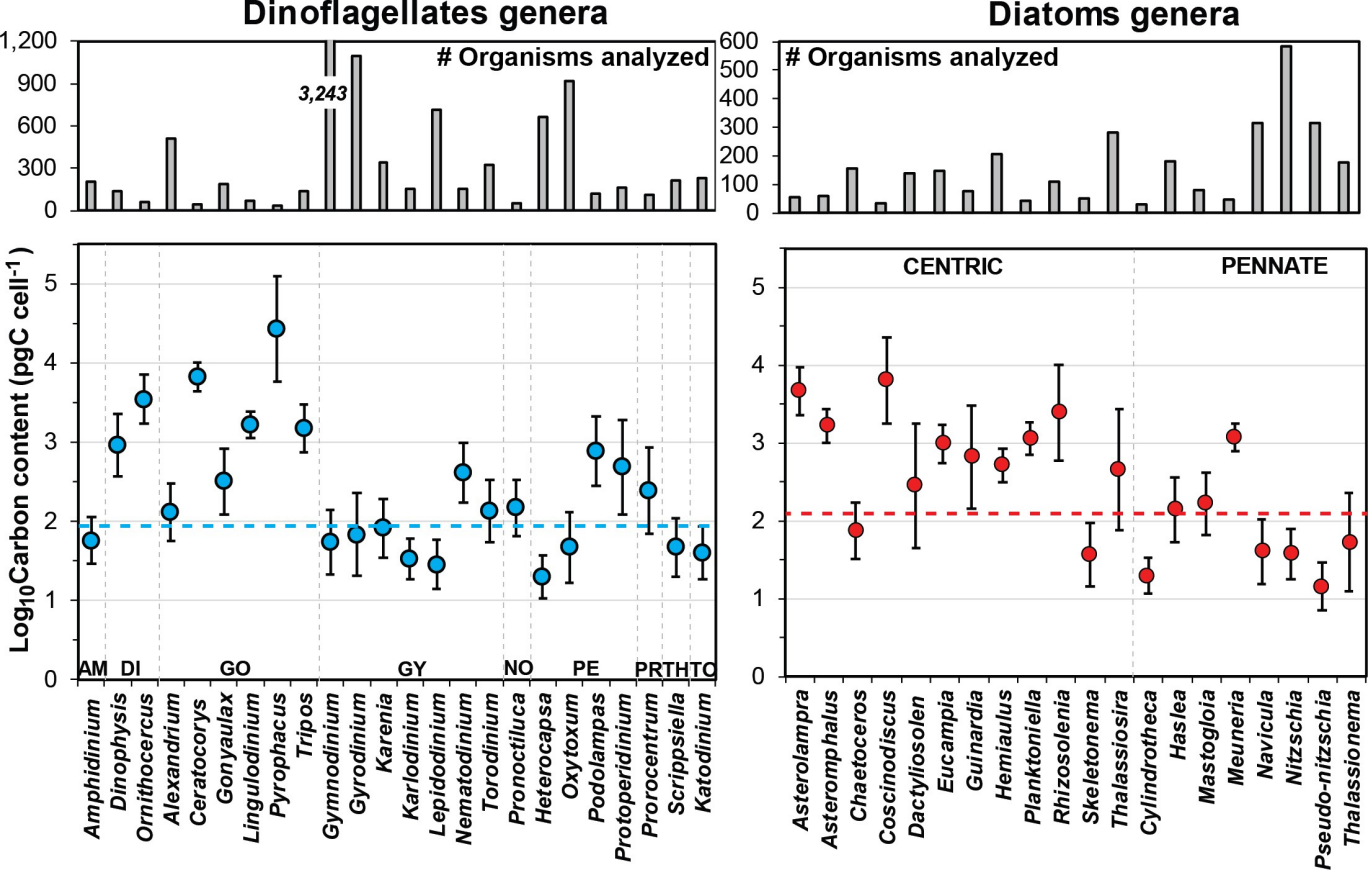
Logarithmic (Log_10_) mean (±SD) of carbon content (pg C cell^-1^) for phytoplankton genera. Average log-values of carbon per cell (pg C cell^-1^; lower panels) for genera of dinoflagellates (blue points) and diatoms (red points). Only taxa with more than 30 cell measurements per genus were included. Grey bars in the upper panels indicate the number of organisms analyzed for each genus. Blue and red dashed lines represent average carbon per cell of total dinoflagellates and diatoms, respectively. Dinoflagellate orders: AM, Amphidiniales; DI, Dinophysales; GO, Gonyaulacales; GY, Gymnodiniales; NO, Noctilucales; PE, Peridiniales; PR, Prorocentrales; TH, Thoracosphaerales; TO, Tovelliales.

According to the log-format of data, differences of up to 3 orders magnitude can be observed in mean carbon content within genera belonging to ten dinoflagellate orders and two diatom shapes (Fig 6 and S2 Fig). Overall, total diatoms had a higher cell carbon content than total dinoflagellates, with average values of 135 and 80 pg C cell^-1^, respectively (averages back-transformed from logarithmic data). Around these means, a wide range of carbon content values is observed among the genera commonly found in this ecosystem (Fig 6). Genera belonging to Dinophysales and Gonyaulacales show carbon content values mostly above the overall dinoflagellate average. The same case is observed for centric diatoms. For instance, *Pyrophacus* (Gonyaulacal dinoflagellate, Fig 3B) and *Coscinodiscus* (centric diatom, Fig 4A) showed the highest average values of carbon per genera (26,866 and 6,386 pg C cell^-1^, respectively). In contrast, pennate diatoms and some dinoflagellate genera in the orders Gymnodiniales, Peridiniales, Thoracosphaerales, and Tovelliales, display carbon content values closer to or below the overall average per group. Such is the case of *Heterocapsa* (Peridinial dinoflagellate) and *Pseudo-nitzschia* (pennate diatom), which had a carbon content 3 orders of magnitude lower (20 and 14 pg C cell^-1^, respectively) relative to other genera (Fig 6). The most common cell measurements in samples corresponded to Gymnodiniales (62% of total dinoflagellates; Table 1), and particularly from ~3,000 cells of *Gymnodinium*, which accounted for ~50% of the measurements performed to this order. However, given the flattened shape and small body size (~15 μm length), its carbon content per cell was notably low (54 pg C cell^-1^). Among diatoms, *Nitzschia* (Fig 4F) was the genus measured most frequently (~600 cells, 30% of total pennate measurements) and also had a low carbon content (37 pg C cell^-1^) (Fig 6).

**Fig 7:**
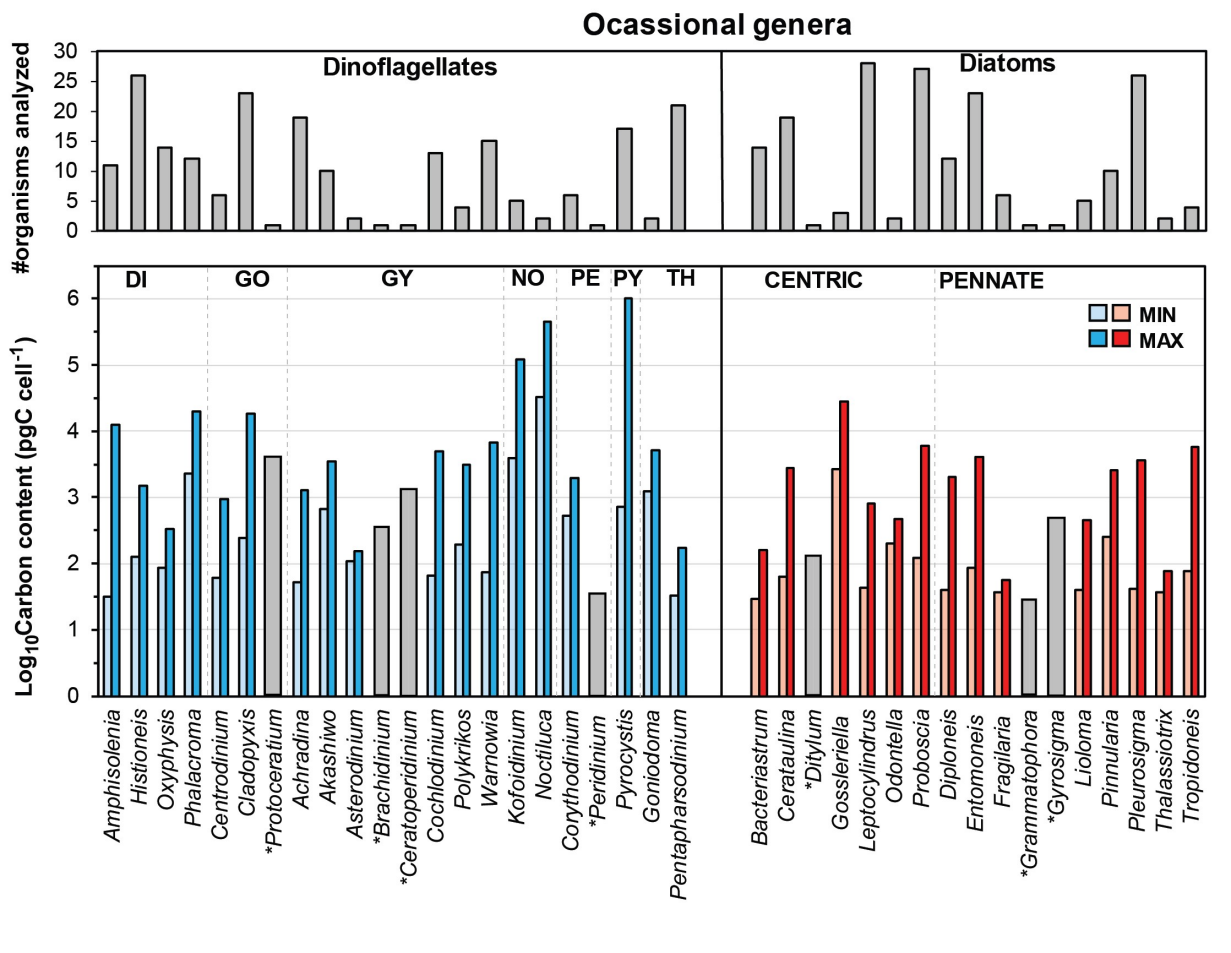
Logarithmic (Log_10_) minimum and maximum values of carbon content (pg C cell^-1^) for ocassional genera. Minimum (light-colored bars) and maximum (dark-colored bars) log-values of carbon content (pg C cell^-1^; lower panels) for genera of dinoflagellates (blue bars) and diatoms (red bars) occasionally found in phytoplankton samples (i.e., <30 cells per genus). Grey bars in the upper panels indicate the number of organisms analyzed per genus. Asterisks denote genera with only a single cell measurement for the carbon content estimation (grey bars in the lower panels). Dinoflagellate orders: DI, Dinophysales; GO, Gonyaulacales; GY, Gymnodiniales; NO, Noctilucales; PE, Peridiniales; PY, Pyrocystales; TH, Thoracosphaerales.

Genera occasionally found in samples (22 dinoflagellates and 17 diatoms) provided a low number of cell measurements (i.e., <30 individuals; Table 1) that precluded robust average computations. However, minimum and maximum values are considered to give a rough idea of how significant in carbon terms these rare taxa can be within the SGoM ecosystem (Fig 7). In this context, the carbon content of the Noctilucal dinoflagellates *Noctiluca* and *Kofoidinium* is notably high, with values of up to 450,000 pg C cell^-1^ (Fig 7). Although these two heterotrophic and unarmored genera were occasionally found in this study, some individuals showed large dimensions (e.g., >200 μm in diameter for *Noctiluca*); hence, only a few cells per liter could be suffient to yield significant carbon biomass for these organisms. Large specimens of *Noctiluca scintillans* (~300–2,000 μm in diameter) have been previously reported in coastal waters of Veracruz and Tabasco [3] and in continental shelf waters of the Yucatan Peninsula [15]. Besides, considering the important role of this species in other marine ecosystems as a consumer of some potentially toxic algal blooms [45], its carbon content and biomass estimated in this work may be useful for trophic ecology studies in SGoM waters.

Considering the large variability in carbon content estimates among the dinoflagellate and diatom genera (Figs 6 and 7), our values are roughly in agreement with other carbon factors found in open-ocean ecosystems. For instance, during the JGOFS time-series study conducted in oceanic waters of the northeast Atlantic Ocean, nano-sized heterotrophic dinoflagellates with carbon content values between 32 and 51 pg C cell^-1^ (mostly *Gyrodinium* type, *Katodinium*) were the dominant fraction in the group [46]. However, the specific-species variability comprising the genera reported across several ecosystems and, occasionally, the different methodologies reported in the literature, make such comparisons difficult. For example, in contrast with our low estimates of carbon per cell, a large fraction (>40%) of 113 diatom and dinoflagellate taxa analyzed in an extensive area of North Atlantic waters recorded carbon content values between 1,000 and 10,000 pg C cell^-1^ and higher (>10,000 pg C cell^-1^) in dinoflagellates [47]. However, that investigation not only covered several hydrographic regimens from subpolar to near-subtropical waters, but also phytoplankton cells were captured with a Continuous Plankton Recorder through a large sampling mesh (270 μm on a side); hence, small cells of diatoms and dinoflagellates were likely undersampled relative to larger ones [47]. For this reason, we consider that this information is specific, variable, and useful only for the region evaluated. Also, it can be reliably compared between phytoplankton groups and marine ecosystems when carbon estimates are used for computations of biomass standardized by volume. Carbon biomass assessments for SGoM oceanic waters will be discussed below.

### Accuracy of average biovolume and carbon content assessments

The cells of dinoflagellates and diatoms included in the carbon content averages computed in this study comprising more than 30 organisms per genus (Fig 6) yielded a standard error (SE) ≤5% of the mean (S1 Fig). Even for Pyrocystal dinoflagellates represented by *Pyrocystis* in this study (Table 1, S2 Fig), SE was 6% of the mean for carbon computations of 17 cells only. Other genera, such as the diatoms *Dactyliosolen* and *Thalassionema*, had an error coefficient of 7%, but only when the first 30 cells measured are considered. Instead, the total number of measurements for these genera (141 and 175 cells, respectively) yields a SE ≤3% of the carbon mean (S1 Fig) and agrees with the recommendation of [33] about taking as many measurements as possible to reduce the SE. According to [48], at least 25 cells should be measured to calculate the volume from which the average BV is derived for each species. Similarly [19], also found that 25 measurements of linear dimensions would be sufficient to obtain a SE <10% of the mean for four diatom species. Thus, we consider that our carbon content averages were derived from a reasonable number of cell measurements (i.e., ≥30 cells per genus), yielding an error coefficient ≤4% for all genera included. These values are also based on a careful choice of appropriate volumetric shapes for all dinoflagellate and diatom genera found in the samples (S1 Table). This selection is a key factor to obtain accurate biovolume results, which determine the variability in carbon-based biomass patterns for these organisms. In this regard, some authors stress the importance of producing a more complex geometric model for some species with combined geometric shapes (e.g., *Tripos*), especially if they are dominant in phytoplankton assemblages [33]. Even for some geometric shapes, BV calculations require measuring the third dimension (cell thickness), which can be limited by the microscope image processing that provides only two dimensions. Depending on the body shapes of the genera, the third, "hidden dimension" (HD), in this study is assumed to be a fraction (50–89%) or equal to cell diameter (S1 Table). The proportions took into account some aspect ratios (L:W:depth ratio) determined from a subset of cells measured (as in [2]), and also from approximations reported in [20] for some genera of dinoflagellates and diatoms (e.g., *Gymnodinium spp*., HD = 67% of W) and from the Marine Ecosystem Data (MAREDAT), a global database for diatoms [22].

Another aspect that may affect the reliability of our determinations is the impact of widely used preservative solutions (e.g., acid Lugol’s iodine, Bouin’s solution, buffered formaldehyde, or glutaraldehyde), on cell biovolume of marine planktonic organisms (and hence, on cell carbon content estimates) [49–51]. In particular, the effects of fixation on the biovolume of diatoms and dinoflagellates has been described in the literature, including cell swelling and shrinking by acid Lugol and glutaraldehyde preservatives [51]. However, this effect is highly variable across species because it depends on factors such as fixative type and strength or sample storage time, which can determine the magnitude and direction of the species-specific cell volume alteration and, in turn, can lead to biases in biomass estimates of individual species. Therefore, in samples composed of several mixed species of these phytoplankton groups (as in this study), the effects of preservatives are seemingly not significant when assessing carbon biomass [51]. Besides, the carbon:BV equations used in this study contemplate the fixation effects on carbon content estimates since they are based on live and fixed-cell volume data [2].

In conclusion, our BV estimates can be considered reliable as these are based on a fairly large number of cells measured and several geometric approximations per genus, thus yielding robust estimators of mean carbon content (SE ≤4%). Besides, these estimates were obtained for several mixed species of dinoflagellates and diatoms, thus minimizing the specific-species effects of preservatives on cell size and body shape.

### Temporal and regional carbon biomass assessments

**Fig 8:**
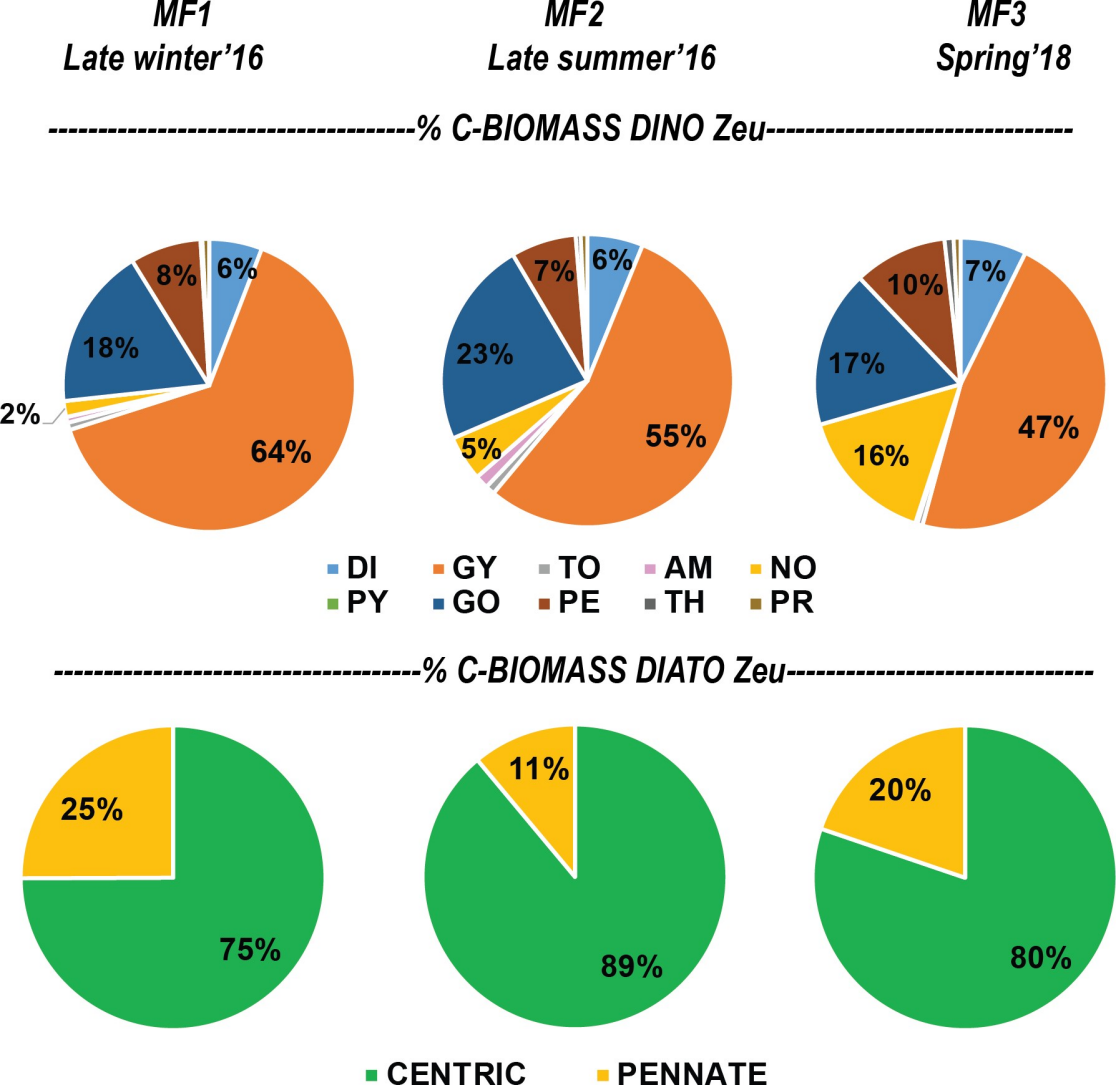
Relative per-cruise percentage of depth-integrated carbon biomass for dinoflagellate orders and diatom shapes. Carbon biomass contribution (%) of dinoflagellate orders and diatom shapes in the euphotic zone (Zeu: surface to ~150 m depth) during MF1 (late winter 2016), MF2 (late summer 2016), and MF3 (spring 2018) cruises. Dinoflagellate orders: DI, Dinophysales; GY, Gymnodiniales; TO, Tovelliales; AM, Amphidiniales; NO, Noctilucales; PY: Pyrocystales; GO, Gonyaulacales; PE, Peridiniales; TH, Thoracosphaerales; PR, Prorocentrales. Diatom shapes: CENTRIC and PENNATE.

The cell carbon estimates reported in this study are considered valuable information for carbon biomass assessments and relative contributions of the main taxa within the phytoplankton community. For instance, the most frequent cell measurements and also the most abundant organisms within the euphotic zone during MF1 (late winter 2016), MF2 (late summer 2016), and MF3 (spring 2018) cruises belonged to Gymnodinial dinoflagellates and pennate diatoms (Tables 1 and 2). Both were represented mostly by small-sized and low-carbon-content genera, such as the unarmored dinoflagellates *Gymnodinium* and *Gyrodinium*, and the diatoms *Nitzschia* and *Navicula* (Fig 6). These genera have been frequently recorded in different seasons of the year in several regions of the SGoM ecosystem [7, 8, 11, 15, 18, 31]. Although both groups have a low carbon content per cell (S2 Fig), the high abundance of Gymnodiniales made a significant contribution to total dinoflagellate biomass (47–64% of total carbon), while pennate diatoms only made as much as 25% of total diatom biomass (Fig 8). Instead, the highest carbon fraction of diatoms was made of centric shapes since their large-sized genera (e.g., *Coscinodiscus*, Fig 4A) have predominantly high carbon content per cell (Figs 6 and 8). Therefore, Gymnodinial dinoflagellates and centric diatoms represented a significant part of the nano- and microplankton biomass community in SGoM waters.

**Table 2:** Per-cruise abundance (cells L^-1^) and carbon biomass (μg C L^-1^) of dinoflagellates and diatoms.

		MF1			MF2			MF3		
**ABUND. (cells L**^**-1**^**)**		***Median***	***Min***	***Max***	***Median***	***Min***	***Max***	***Median***	***Min***	***Max***
**TOTAL**	**DINO**	2,009	56	15,706	2,690	14	12,104	2,724	46	16,621
	**DIATO**	209	16	2,240	308	4	102,067	470	30	5,680
**ORDER**	**DY**	20	0	220	20	0	186	30	0	220
	**GY**	1,605	48	13,278	1,970	10	9,298	1,880	34	13,611
	**TO**	30	0	460	62	0	665	30	0	260
	**AM**	4	0	486	53	0	549	4	0	90
	**NO**	8	0	216	4	0	112	10	0	90
	**PY**	0	0	20	0	0	28	0	0	30
	**GO**	25	0	180	62	0	813	110	0	380
	**PE**	190	0	1,722	247	0	2,265	450	2	2,822
	**TH**	4	0	160	20	0	610	50	0	320
	**PR**	0	0	610	4	0	301	10	0	65
**SHAPE**	**CEN**	38	0	740	80	0	90,801	100	0	3,280
	**PEN**	149	4	1,789	217	4	17,843	370	30	2,400
**BIOM. (μgC L**^**-1**^**)**		***Median***	***Min***	***Max***	***Median***	***Min***	***Max***	***Median***	***Min***	***Max***
**TOTAL**	**DINO**	0.207	0.013	1.502	0.315	0.001	5.465	0.325	0.007	2.263
	**DIATO**	0.024	0.001	0.380	0.083	0.000	63.885	0.083	0.003	1.065
**ORDER**	**DY**	0.006	0.000	0.098	0.014	0.000	0.257	0.014	0.000	0.117
	**GY**	0.125	0.004	1.004	0.151	0.001	1.317	0.150	0.002	0.956
	**TO**	0.001	0.000	0.018	0.002	0.000	0.026	0.001	0.000	0.010
	**AM**	0.000	0.000	0.028	0.003	0.000	0.032	0.000	0.000	0.005
	**NO**	0.001	0.000	0.488	0.001	0.000	1.267	0.001	0.000	1.217
	**PY**	0.000	0.000	0.373	0.000	0.000	0.523	0.000	0.000	0.556
	**GO**	0.013	0.000	0.852	0.054	0.000	4.637	0.054	0.000	0.900
	**PE**	0.014	0.000	0.154	0.021	0.000	0.287	0.029	0.000	0.171
	**TH**	0.000	0.000	0.010	0.001	0.000	0.061	0.002	0.000	0.015
	**PR**	0.000	0.000	0.148	0.001	0.000	0.073	0.002	0.000	0.016
**SHAPE**	**CEN**	0.012	0.000	0.313	0.066	0.000	61.729	0.066	0.000	0.962
	**PEN**	0.008	0.000	0.242	0.012	0.000	2.156	0.018	0.003	0.344

Median, minimum, and maximum abundance (cells L^-1^) and carbon biomass (μg C L^-1^) in the euphotic zone (surface to ~150 m depth) of dinoflagellate orders and diatom shapes during MF1 (late winter 2016), MF2 (late summer 2016), and MF3 (spring 2018) cruises. Dinoflagellate (DINO) orders: DI, Dinophysales; GY, Gymnodiniales; TO, Tovelliales; AM, Amphidiniales; NO, Noctilucales; PY: Pyrocystales; GO, Gonyaulacales; PE, Peridiniales; TH, Thoracosphaerales; PR, Prorocentrales. Diatom (DIATO) shapes: CEN: Centric; PEN: Pennate.

Additionally, several of the dinoflagellates analyzed in this work have significant ecological roles in marine ecosystems. Thus, for example, many mixotrophic and heterotrophic genera belonging to the athecate Gymnodinoids such as *Gymnodinium*, *Gyrodinium*, *Polykrikos*, and armored forms like *Protoperidinium*, have diverse feeding mechanisms, and hence substantially impact the daily biomass of primary producers or even other heterotrophic protists and metazoans, including heterotrophic bacteria [52, 53]. In the northern Gulf of Mexico, *Gyrodinium* has been observed ingesting chain-forming diatoms and is described as a major component of microzooplankton [54]. Although the taxonomic analysis in this study did not fully allow classifying all the dinoflagellate genera measured and counted in each cruise according to trophic mode, a substantial fraction of carbon biomass (~70%) can be attributed to mixotrophic and heterotrophic genera based on descriptions in the literature [47, 55, 56]. This observation suggests that carbon biomass produced in the euphotic zone by diatoms or other autotrophic cells might be transferred to higher trophic levels through these nano- and micro-sized dinoflagellate genera; hence, they are likely to play a major role as consumers within food webs in SGoM ecosystem. Besides, some frequent dinoflagellate species in SGoM waters found in this study are known or presumed to be toxic, such as *Dinophysis caudata*, *Protoperidinium oblongum*, *Lingulodinium polyedra*, *Dinophysis rapa*. Some, like *Karenia brevis*, are notable HAB species that cause severe impacts on marine ecosystems, public health, and the regional economy [12, 57, 58]. Therefore, the morphometric information and carbon-biomass assessments set the basis to define some key functional groups for biogeochemical model parameterizations in this oceanic region to understand the functioning of the gulf ecosystem.

**Fig 9:**
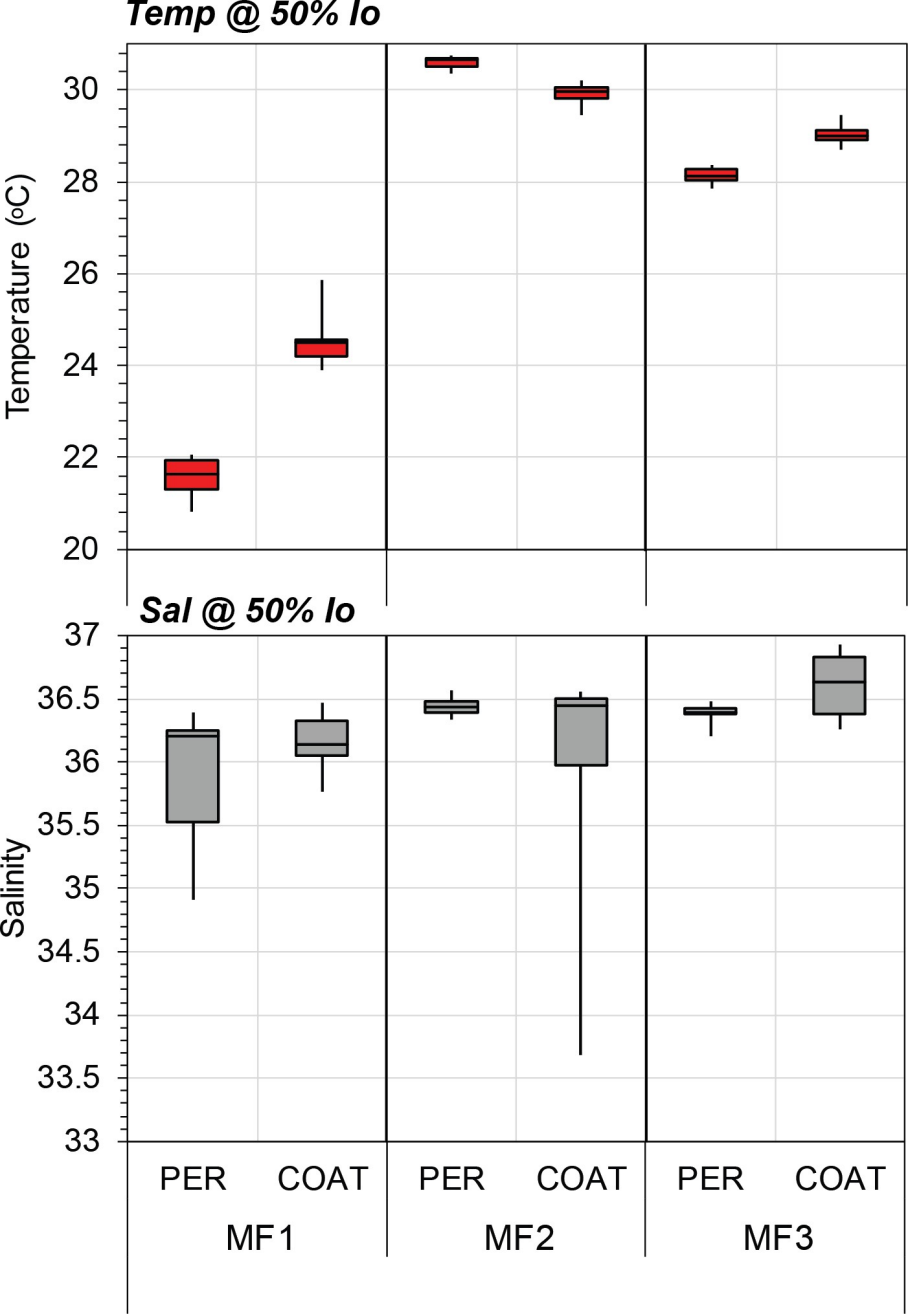
Temperature (°C) and salinity per cruise in the upper euphotic zone. Median values of temperature (°C; upper panel) and salinity (lower panel) from data recorded between 11–17 m depth (~50% surface irradiance, %Io) at the Perdido (PER) and Coatzacoalcos (COAT) regions during MF1 (late winter 2016), MF2 (late summer 2016), and MF3 (spring 2018) cruises. For each hydrographic variable, the rectangular box represents the middle 50% of the data, delimited by the lower quartile (Q1) and upper quartile (Q3). The median is represented by a straight line inside the box. Whiskers are drawn for the minimum and maximum values.

**Fig 10:**
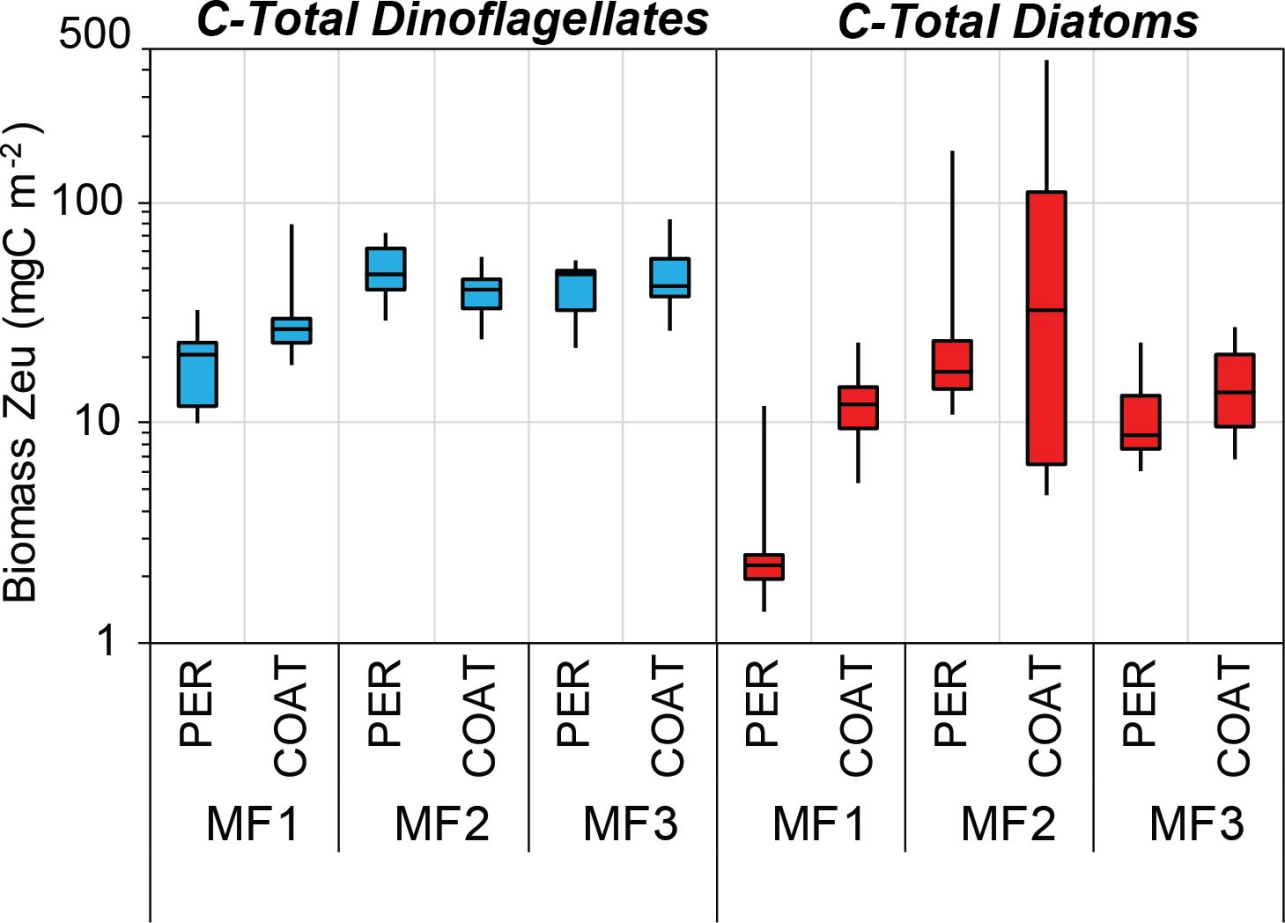
Depth-integrated per-cruise carbon biomass (mg C m^-2^) of total dinoflagellates and diatoms. Median values of integrated carbon biomass (mg C m^-2^) of total dinoflagellates (blue box-plots) and diatoms (red box-plots) in the euphotic zone (surface to ~150 m depth) at the Perdido (PER) and Coatzacoalcos (COAT) regions during MF1 (late winter 2016), MF2 (late summer 2016), and MF3 (spring 2018) cruises. The rectangular box represents the middle 50% of the data for each group, delimited by the lower quartile (Q1) and upper quartile (Q3). The median is represented by a straight line inside the box. Whiskers are drawn for the minimum and maximum values. Y-axis is logarithmically scaled.

Morphological traits such as geometric shapes and cell sizes have been recognized as useful descriptors of the ecological status in aquatic ecosystems, rather than taxonomic descriptors [21, 43]. They also regulate features to determine taxonomic diversity [59] and the seasonal distribution of phytoplankton in marine ecosystems [60]. Similarly, the cell carbon content of dinoflagellates and diatoms has also been considered to be a key functional trait in the seasonal succession and spatial variation of the phytoplankton community structure [47]. Thus, the cell-size and carbon-per-cell data obtained in the current study provide valuable sources of information to better understand the ecological dynamics of dinoflagellates and diatoms in the ecosystem. For instance, spatial and temporal changes in the abundance and composition of these groups attributed to regional hydrographic conditions in SGoM waters [7, 11] could also be due to variations in their size classes and carbon content shaped by physiological differences between and within these microalgae groups related to nutrient requirements, uptake, and cell growth [41]. In this study, seasonal and regional variability is observed in the hydrographic conditions and carbon biomass of dinoflagellates and diatoms within the euphotic zone during late winter (MF1), late summer (MF2), and spring cruises (Figs 9 and 10). Based on the changes in abundance in both groups and the structure of cell carbon content, biomass variability can be associated with temporal and spatial changes of the genera making the total biomass, likely regulated by the environmental forcing in each cruise. For example, the higher biomass of dinoflagellates in MF1 (Fig 10) was attributed mostly to the high abundance (>70% of total abundance; Table 2) of small-sized genera (i.e., carbon content <100 pg C cell^-1^) in the Gymnodinial order. During MF2 and MF3, in contrast, patchiness of high dinoflagellate biomass (>1 μg C L^-1^) was observed within the euphotic zone and attributed to the low abundance (<4% of total) of large-sized cells (i.e., carbon content >10,000 pg C cell^-1^) belonging to Noctilucales (e.g., *Kofoidinium* and *Noctiluca*), Pyrocystales (e.g., *Pyrocystis*), and Gonyaulacales (e.g., *Pyrophacus*) (Table 2). To note, the occasional occurrence of large-sized Noctilucal dinoflagellate genera like *Kofoidinium* increased the relative fraction of this order to total biomass in MF2 and MF3 (Fig 8), when the seasonal warming in summer and spring was evident in the upper euphotic zone (<20 m depth; Fig 9). The contribution of high-carbon-content genera such as *Kofoidinium* to total dinoflagellate abundance has been observed mainly during spring-summer within SGoM waters [10, 18]. These results evidence the prevalence of tropical, heterotrophic, and high-carbon-content genera during warmer and more stratified hydrographic conditions, suggesting a succession of smaller-sized species followed by larger-sized ones as environmental conditions change from cold (i.e., MF1) to warm (i.e., MF2 and MF3).

**Fig 11:**
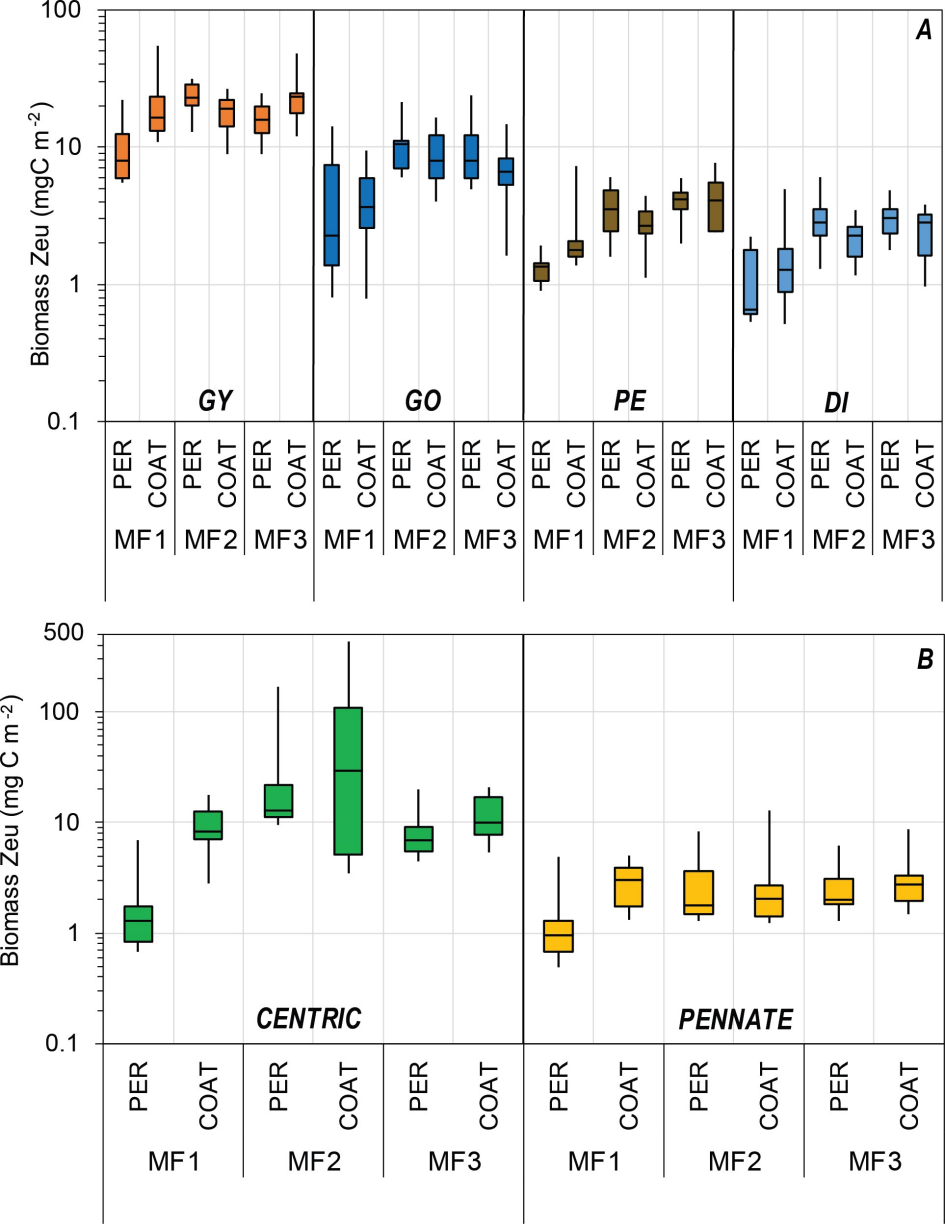
Depth-integrated per-cruise carbon biomass (mg C m^-2^) of dinoflagellate orders and diatom shapes. Median values of integrated carbon biomass (mg C m^-2^) in the euphotic zone of (A) dinoflagellate orders contributing 75% or more to total biomass (box plots at the upper panel) and (B) centric and pennate diatoms (green and yellow box-plots at the lower panel, respectively) at the Perdido (PER) and Coatzacoalcos (COAT) regions during MF1 (late winter 2016), MF2 (late summer 2016), and MF3 (spring 2018) cruises. The rectangular box represents the middle 50% of the data for each group, delimited by the lower quartile (Q1) and upper quartile (Q3). The median is represented by a straight line inside the box. Whiskers are drawn for the minimum and maximum values. Different logarithmic scales in the Y-axis are used for dinoflagellates and diatoms. Dinoflagellate orders: GY, Gymnodiniales (orange box-plots); GO, Gonyaulacales (dark blue box-plots); PE, Peridiniales (brown box-plots); DI, Dinophysales (light blue box-plots).

Overall, the distribution range of depth-integrated carbon biomass values in the euphotic zone for each cruise and region was more consistent for dinoflagellates versus diatoms (Fig 10). The lowest biomass (10.0 mg C m^-2^) for dinoflagellates was estimated in MF1 at Perdido, where the median value (20.3 mg C m^-2^) was about 2 times lower than those observed at the same region in MF2 and MF3. High depth-integrated biomasses were found in MF2 and MF3, with a peak of 84.1 mg C m^-2^ at Coatzacoalcos in MF3 (Fig 10). The increase in dinoflagellate biomass toward warmer seasons is consistent with their dominance reported in SGoM waters in summer months, when environmental conditions are favorable [10, 18, 31]. It can also be associated with the detached Loop Current eddies (LCEs) propagating to the western basin [34], which are likely transporting Caribbean species into gulf waters. Concerning tropical intrusion, the transport of pico-phytoplankton populations, particularly the low-light *Prochlorococcus* ecotypes trapped inside LCEs in summer, was recently evidenced in SGoM waters [61]. Hence, it is reasonable to assume that the peak biomass of dinoflagellates recorded during MF2 at Perdido (Fig 10) were likely influenced by the arrival of warmer eddies to this region. In fact, a large LCE (Poseidon Eddy) drifting westward (~24–27°N) was tracked from April to November 2016 [62], so this anticyclonic structure likely kept some dinoflagellates trapped (with continued growth) along its journey to the west. Thus, a large fraction of total dinoflagellate biomass during MF2 can be attributed to the most abundant and frequent genera like Gymnodinales and large-carbon-content genera like Gonyaulacales (Fig 11A, Table 2), likely modulated by the physical dynamics of the sampling period.

Despite the high variability of up to 2 orders of the magnitude observed among cruises and regions for diatom biomass values, this group generally displayed median estimates lower than those of dinoflagellates (Fig 10). Total diatoms had its lowest median depth-integrated biomass (2.3 mg C m^-2^) in Perdido during MF1, while the highest (32.2 mg C m^-2^) was observed in Coatzacoalcos during MF2 (Fig 10). As regards diatom shapes, peak biomass values (>20 mg C m^-2^) were attributed mainly to centric shapes, while the lowest (<2 mg C m^-2^) were related to pennate shapes (Fig 11B). Particularly during MF2, large cylindrical cells dominated diatom biomass and increased the total biomass (up to 438 mg C m^-2^) in the euphotic zone. This enhanced biomass was attributed to a bloom of the centric diatoms *Dactyliosolen fragilissimus* and *Rhizosolenia spp*. in the upper layers (<20 m depth) in stations influenced by continental runoff, as evidenced by the minimum salinity records (~33) in the Coatzacoalcos region (Table 2, Fig 9). Large freshwater runoff from the Coatzacoalcos river and Grijalva-Usumacinta system is usually seen during the rainy season (summer-autumn), stretching beyond the continental platform, due to cross-shelf transports produced by the confluence of along-shelf currents in the SGoM [36, 37]. This circulation pattern is important because it likely carries nutrient-rich waters onto the shelf, favoring phytoplankton growth, and the shelf may in turn transport those more productive waters into the deep ocean. A seasonal peak of offshore cross-shelf transport at the southern Bay of Campeche has been reported to occur during October–November, evidenced by the high chlorophyll-*a* values estimated from satellite images [63]. Chlorophyll-rich waters were observed during late summer 2016 at the Coatzacoalcos region, contrasting with the late winter 2016 and spring 2018 periods (S3 Fig). Therefore, our biomass estimates are influenced by the density of the dominant groups within the euphotic zone, the composition of body shapes, and the environmental variability of waters inhabited by diatoms and dinoflagellates.

From a broader perspective, the range of carbon biomass values for dinoflagellates and diatoms assessed in our study is roughly in agreement with estimates for both global and open-ocean ecosystems reported in the literature [22, 46, 64–66]. Global assessments of integrated carbon biomass in the euphotic zone (down to 100 m depth) performed for diatoms have shown values between 10 and 100,000 mg C m^-2^, which encompass a large variety of ecosystems [22]. Similarly, global diatom biomass estimates reported for the upper 200 m cover a wide range of values (0–4,150 μg C L^-1^) with a median of about 1.74 μg C L^-1^, which is slightly lower (1.3 μg C L^-1^) in open-ocean waters [64]. A significant fraction of our diatom biomass values fall close to the lower limit of these global estimates. However, in this vast effort to estimate diatom carbon biomass from data obtained in different oceans, Gulf of Mexico waters were poorly represented [22, 64]. For this reason, the carbon biomass assessment for diatoms conducted in our study can be considered adequate and consistent with the low abundances and the predominance of low-carbon-content cells within the oceanic SGoM ecosystem. For dinoflagellates, our biomass values are more comparable with other open-ocean ecosystems. From five years of observations at the ALOHA station in the North Pacific Subtropical Gyre, the depth-integrated abundance and carbon biomass of eukaryotic phytoplankton groups, including dinoflagellates and diatoms, were seasonally variable within the euphotic zone (0–200 m depth). Biomass values in this study oscillated roughly between 5–40 mg C m^-2^ (the middle 50% of the data) throughout the year and were more variable and higher for diatoms vs. autotrophic dinoflagellates [65]. Besides, heterotrophic dinoflagellates generally showed biomass values <50 mg C m^-2^ in this open-ocean ecosystem [65]. From carbon estimates obtained for heterotrophic dinoflagellates and ciliates in oligotrophic subtropical Sargasso Sea waters, on average half of the biomass for both groups was dominated by cells >20 μm of heterotrophic dinoflagellates, ranging from 0.1 to 2.1 μg C L^-1^ within the first 150 m depth [66]. As regards cell size, during the spring bloom in the northeast Atlantic Ocean, nano-sized heterotrophic dinoflagellates represented about 77–80% of total carbon biomass, with values that fluctuated between 0.1 and 3.1 μg C L^-1^ in the upper 200 m [46]. Thus, small-sized cells with significant numerical occurrence (e.g., *Gymnodinium*) and large-sized cells with low abundances (e.g., *Pyrophacus*) can contribute similarly to total dinoflagellate biomass, depending on the environmental circumstances that may favor one or the other type.

Compared with other phytoplankton components, the carbon biomass of diatoms and dinoflagellates seems to represent a small fraction in open-ocean SGoM waters. Instead, the pico-phototroph community composed of cyanobacteria populations (*Prochlorococcus* and *Synechococcus*) and pico-eukaryote assemblages can reach biomass values one order of magnitude higher than these pelagic microalgae. During winter, a biomass average (± SD) of 24.57 ± 7.62 μg C L^-1^ has been reported for pico-phytoplankton populations living within the euphotic zone of SGoM oceanic waters [67]. This mean value decreased as warmer spring/summer conditions developed (6.3 ± 4.1 μgC L^-1^) and was dominated by *Prochlorococcus* populations [61]. These values suggest that dinoflagellates and diatoms do not contribute substantially to the total algal community, at least in the oceanic region. However, under particular oceanographic, hydrological, and atmospheric conditions of the different regions across this extensive basin (e.g., cross-shelf exchange modulated by the interaction of mesoscale eddies with the western GoM shelf; [68]), large amounts of carbon in both groups can be transported from productive regions on the continental shelves to offshore waters (e.g., diatom-dominated surface waters at the Coatzacoalcos region in MF2), or high-carbon-content components may intrude from Caribbean regions inside warmer eddies (e.g., large-sized cells of Noctilucales and Gonyaulacales at the Perdido region in MF2). Therefore, the biomass of dinoflagellates and diatoms can be highly significant under particular conditions in oceanic SGoM waters, hence contributing to a greater extent than the background carbon of picoplankton populations.

## Conclusions

This work represents a reliable source of information required for ecological and biogeochemical studies for open-ocean SGoM waters, a region poorly understood as regards the standing stocks and biogenic carbon pathways regulating the carbon cycle in the oceans. This extensive ecosystem harbors a wide diversity of marine species and a high biomass of fish (many commercially important), seabirds, and marine mammals, sustained by moderate primary productivity [23]. Thus, the knowledge of the contribution, in carbon terms, of some autotrophic components advances our understanding of the functional role of diatoms and dinoflagellates within pelagic food webs in this oligotrophic region. Given the current global climate-change context, the linkages between these plankton groups at the base of trophic webs, their zooplankton predators, and larval fish are crucial for evaluating the ecological, economic, and social impacts on this open-ocean ecosystem.

## Supporting information

S1 Table Geometric shapes and equations used for calculating cell biovolume (V) of the dinoflagellates and diatoms genera in the SGoM. Phytoplankton samples were collected from two oceanic regions (Perdido and Coatzacoalcos) during four cruises conducted in late winter (MF1), late summer (MF2), and spring (MF3 and MF4). The equations indicate two linear dimensions measured under the microscope for each genus. In some cases, the third dimension is based on some known measure, according to average data from Olenina et al. (2006) and Leblanc et al. (2012). The abbreviations used in each equation according to their references are: Olenina et al. (2006): *d* = diameter (subscripts 1 and 2 refer to the large and small diameter, respectively), *h* = height, *l* = length, *w* = width; Hillebrand et al. (1999): *a* = apical axis (length), *b* = transapical axis (width), *c* = pervalvar axis (height), *d* = diameter, *h* = height, *l* = length of one side, *m* = height of a triangle; Vadrucci et al. (2013): *a* or *d* = diameter, *b* = depth, *h* = height, *z* = height of cone; Sun and Liu (2003): *a* = length, *b* = width.
(PDF)

S1 Fig Error coefficients of logarithmic carbon content data for common genera of two phytoplankton groups. The standard error (SE) expressed as percentage of the mean for genera of dinoflagellates (left panels) and diatoms (right panels) plotted in function of the total number of cells measured per genus. Inset panels show error coefficients from the first 50 cells measured. Dinoflagellate orders: AM, Amphidiniales; DI, Dinophysales; GO, Gonyaulacales; GY, Gymnodiniales; NO, Noctilucales; PE, Peridiniales; PR, Prorocentrales; PY, Pyrocystales; TH, Thoracosphaerales; TO, Tovelliales.
(PDF)

S2 Fig Logarithmic (Log10) mean (±SD) of carbon content (pg C cell^-1^) for two phytoplankton groups. Average log-values of carbon per cell (pg C cell^-1^; lower panels) for dinoflagellate orders (blue points) and diatom shapes (red points). The total number of genera per dinoflagellate order and diatom shape is indicated at the upper panels (black bars). *Dinoflagellate order with less than 30 individuals.
(PDF)

S3 Fig Satellite images of chlorophyll-*a* concentration (CHL; mg m^-3^) in the Gulf of México. Average images of cruise-mean daily mapped chlorophyll-*a* concentration (CHL; mg m^-3^) for (A) late winter 2016 (MF01), (B) late summer 2016 (MF02) and (C) spring 2018 (MF03). Overlaid vectors indicate geostrophic currents as derived from the cruise-mean maps of sea level anomaly (MSLA). Sea color and MSLA images were obtained from the Copernicus Marine Environment Services server (marine.copernicus.eu). For each cruise, sampling stations are indicated as red dots.
(PDF)
