# Supplementary material for: Cell carbon content and biomass assessments of dinoflagellates and diatoms in the oceanic ecosystem of the Southern Gulf of Mexico
Source: PLoS One. 2021 Feb 17;16(2):e0247071. doi: 10.1371/journal.pone.0247071 (PMC7888629; doi:10.1371/journal.pone.0247071)
Supplement: S1 Table — Phytoplankton samples were collected from two oceanic regions (Perdido and Coatzacoalcos) during four cruises conducted in late winter (MF1), late summer (MF2), and spring (MF3 and MF4). The equations indicate two linear dimensions measured under the microscope for each genus. In some cases, the third dimension is based on some known measure, according to average data from Olenina et al. (2006) and Leblanc et al. (2012). The abbreviations used in each equation according to their references are: Olenina et al. (2006): d = diameter (subscripts 1 and 2 refer to the large and small diameter, respectively), h = height, l = length, w = width; Hillebrand et al. (1999): a = apical axis (length), b = transapical axis (width), c = pervalvar axis (height), d = diameter, h = height, l = length of one side, m = height of a triangle; Vadrucci et al. (2013): a or d = diameter, b = depth, h = height, z = height of cone; Sun and Liu (2003): a = length, b = width. (PDF) [file pone.0247071.s001.pdf]

S1 Table. Geometric shapes and equations used for calculating cell biovolume (V) of the dinoflagellates and diatoms genera in the SGoM. Phytoplankton samples were collected from two oceanic regions (Perdido and Coatzacoalcos) during four cruises conducted in late winter (MF1), late summer (MF2), and spring (MF3 and MF4). The equations indicate two linear dimensions measured under the microscope for each genus. In some cases, the third dimension is based on some known measure, according to average data from Olenina et al. (2006) and Leblanc et al. (2012). The abbreviations used in each equation according to their references are: Olenina et al. (2006): *d* = diameter (subscripts 1 and 2 refer to the large and small diameter, respectively), *h* = height, *l* = length, *w* = width; Hillebrand et al. (1999): *a* = apical axis (length), *b* = transapical axis (width), *c* = pervalvar axis (height), *d* = diameter, *h* = height, *l* = length of one side, *m* = height of a triangle; Vadrucci et al. (2013): *a* or *d* = diameter, *b* = depth, *h* = height, *z*= height of cone; Sun and Liu (2003): *a* = length, *b* = width.

# Genus		Geometric shapes	Formula	References
	**Dinoflagellates**<br>**Amphidiniales**			
1	*Amphidinium*	Flattened Ellipsoid	$V = \pi/6 * d_1 * d_2 * h$; ($d_2 = 70\% \ d_1$; from Olenina estimates)	Olenina et al. (2006)
	**Dinophysales**			
2	*Amphisolenia*	Cylinder	$V = \pi/4 * d^2 * h$	Hillebrand et al. (1999); Vadrucci et al. (2013)
3	*Dinophysis*	Flattened Ellipsoid	$V = \pi/6 * d_1 * d_2 * h$; ($d_2 = 77\% \ d_1$; from estimates of this study)	Olenina et al. (2006)
4	*Histioneis*	Prolate spheroid	$V = \pi/6 * d^2 * h$	Hillebrand et al. (1999)
5	*Ornithocercus*	Half sphere	$V = \pi/12 * d^3$	Hillebrand et al. (1999)
6	*Oxyphysis*	Double Cone	$V= \pi/12 * d^2 * z$	Vadrucci et al. (2013)
7	*Phalacroma*	Ellipsoid	$V = \pi/6 * a * b * h$; ($b = 77\% \ a$; same to *Dinophysis*)	Hillebrand et al. (1999); Vadrucci et al. (2013)
	**Gonyaulacales**			
8	*Alexandrium*	Rotational Ellipsoid	$V = \pi/6 * d^2 * h$	Olenina et al. (2006)
9	*Centrodinium*	Double cone	$V= \pi/12 * d^2 * h$	Olenina et al. (2006)
10	*Ceratocorys*	Rotational Ellipsoid	$V = \pi/6 * d^2 * h$	Olenina et al. (2006)
11	*Cladopyxis*	Sphere	$V = \pi/6 * d^3$	Olenina et al. (2006)

12	*Gonyaulax*	Cone with half sphere	$V= \pi/12 * d^2 * h$	Olenina et al. (2006)
13	*Lingulodinium*	Rotational Ellipsoid	$V = \pi/6 * d^2 * h$	Olenina et al. (2006)
14	*Protoceratium*	Rotational Ellipsoid	$V = \pi/6 * d^2 * h$	Olenina et al. (2006)
15	*Pyrophacus*	Prolate spheroid	$V = \pi/6 * d^2 * h$	Hillebrand et al. (1999)
16	*Tripos*	Ellipsoid + 2 cones + 1 cylinder	V = sum of partial volumes	Vadrucci et al. (2013)
	**Gymnodiniales**			
17	*Achradina*	Prolate spheroid	$V = \pi/6 * d^2 * h$	Hillebrand et al. (1999)
18	*Akashiwo*	Prolate spheroid	$V = \pi/6 * d^2 * h$	Hillebrand et al. (1999)
19	*Asterodinium*	Cylinder + 5 cones	V = sum of partial volumes	Hillebrand, et al. (1999)
20	*Brachydinium*	Cylinder + 4 cones	V = sum of partial volumes	Hillebrand, et al. (1999)
21	*Ceratoperidinium*	Prolate spheroid	$V = \pi/6 * d^2 * h$	Hillebrand et al. (1999)
22	*Cochlodinium*	Prolate spheroid	$V = \pi/6 * d^2 * h$	Hillebrand et al. (1999)
23	*Gymnodinium*	Flattened Ellipsoid	$V = \pi/6 * d_1 * d_2 * h$; ($d_2 = 67\% \ d_1$; from Olenina estimates)	Olenina et al. (2006)
24	*Gyrodinium*	Double cone	$V= \pi/12 * d^2 * h$	Olenina et al. (2006)
25	*Karenia*	Flattened Ellipsoid	$V = \pi/6 * d_1 * d_2 * h$; ($d_2 = 79\% \ d_1$; from Olenina estimates)	Olenina et al. (2006)
26	*Karlodinium*	Prolate spheroid	$V = \pi/6 * d^2 * h$	Hillebrand et al. (1999)
27	*Lepidodinium*	Cone with half sphere	$V= \pi/12 * d^2 * h$	Olenina et al. (2006)
28	*Nematodinium*	Prolate spheroid	$V = \pi/6 * d^2 * h$	Hillebrand et al. (1999)
29	*Polykrikos*	Prolate spheroid	$V = \pi/6 * d^2 * h$	Hillebrand et al. (1999)
30	*Torodinium*	Flattened Ellipsoid	$V = \pi/6 * d_1 * d_2 * h$; ($d_2 = 50\% \ d_1$; from Olenina estimates)	Olenina et al. (2006)
31	*Warnowia*	Prolate spheroid	$V = \pi/6 * d^2 * h$	Hillebrand et al. (1999)
	**Noctilucales**			
32	*Kofoidinium*	Sphere	$V = \pi/6 * d^3$	Hillebrand et al. (1999)
33	*Noctiluca*	Sphere	$V = \pi/6 * d^3$	Hillebrand et al. (1999); Olenina et al. (2006)
34	*Pronoctiluca*	Flattened Ellipsoid	$V = \pi/6 * d_1 * d_2 * h$; ($d_2 = 83\% \ d_1$; from Olenina estimates)	Olenina et al. (2006)

	**Peridiniales**			
35	*Corythodinium*	Double cone	$V= \pi/12 * d^2 * h$	Olenina et al. (2006)
36	*Heterocapsa*	Double cone	$V= \pi/12 * d^2 * h$	Olenina et al. (2006); Vadrucci et al. (2013)
37	*Oxytoxum*	Double cone	$V= \pi/12 * d^2 * h$	Olenina et al. (2006)
38	*Peridinium*	Cone with half sphere	$V= \pi/12 * d^2 * h$	Olenina et al. (2006)
39	*Podolampas*	Cone with half sphere	$V= \pi/12 * d^2 * h$	Olenina et al. (2006)
40	*Protoperidinium*	Cone with half sphere	$V= \pi/12 * d^2 * h$	Olenina et al. (2006)
	**Prorocentrales**			
41	*Prorocentrum*	Flattened Ellipsoid	$V = \pi/6 * d_1 * d_2 * h$; ($d_2 = 55\% \ d_1$; from Olenina estimates)	Olenina et al. (2006)
	**Pyrocystales**			
42	*Pyrocystis*	Prolate spheroid	$V = \pi/6 * d^2 * h$	Vadrucci et al. (2013)
	**Thoracosphaerales**			
43	*Goniodoma*	Rotational Ellipsoid	$V = \pi/6 * d^2 * h$	Olenina et al. (2006)
44	*Scrippsiella*	Cone with half sphere	$V= \pi/12 * d^2 * h$	Olenina et al. (2006)
45	*Pentapharsodinium*	Cone with half sphere	$V= \pi/12 * d^2 * h$	Olenina et al. (2006)
	**Tovelliales**			
46	*Katodinium*	Double cone	$V= \pi/12 * d^2 * h$	Olenina et al. (2006)

**#Genus**	**Diatoms**<br>**Centric**			
1	*Asterolampra*	Cylinder	$V = \pi/4 * d^2 * h$	Hillebrand et al. (1999); Vadrucci et al. (2013)
2	*Asteromphalus*	Cylinder	$V = \pi/4 * d^2 * h$	Hillebrand et al. (1999); Vadrucci et al. (2013)
3	*Bacteriastrum*	Cylinder	$V = \pi/4 * d^2 * h$	Hillebrand et al. (1999); Vadrucci et al. (2013)

4	*Cerataulina*	Cylinder	$V = \pi/4 * d^2 * h$	Hillebrand et al. (1999); Olenina et al. (2006); Vadrucci et al. (2013)
5	*Chaetoceros*	Oval cylinder	$V = \pi/4 * d_1 * d_2 * h$; ($d_2$ = 74% d_1; from Olenina estimates)	Olenina et al. (2006)
6	*Coscinodiscus*	Cylinder	$V = \pi/4 * d^2 * h$	Hillebrand et al. (1999); Vadrucci et al. (2013)
7	*Dactyliosolen*	Cylinder	$V = \pi/4 * d^2 * h$	Hillebrand et al. (1999); Vadrucci et al. (2013)
8	*Ditylum*	Prism on triangle-base girdle view	$V = \frac{\sqrt{3}}{4} * a * b^2$	Sun and Liu (2003)
9	*Eucampia*	Oval cylinder	$V = \pi/4 * d_1 * d_2 * h$; ($d_2$ = 85% d_1; from Olenina estimates)	Olenina et al. (2006)
10	*Gossleriella*	Cylinder	$V = \pi/4 * d^2 * h$	Hillebrand et al. (1999)
11	*Guinardia*	Cylinder	$V = \pi/4 * d^2 * h$	Hillebrand et al. (1999); Olenina et al. (2006); Vadrucci et al. (2013)
12	*Hemiaulus*	Cylinder	$V = \pi/4 * d^2 * h$	Hillebrand et al. (1999); Vadrucci et al. (2013)
13	*Leptocylindrus*	Cylinder	$V = \pi/4 * d^2 * h$	Hillebrand et al. (1999); Olenina et al. (2006); Vadrucci et al. (2013)
14	*Odontella*	Oval cylinder	$V = \pi/4 * d_1 * d_2 * h$; ($d_2$ = 50% d_1; from Olenina estimates)	Olenina et al. (2006); Hillebrand et al. (1999)
15	*Planktoniella*	Cylinder	$V = \pi/4 * d^2 * h$	Hillebrand et al. (1999); Vadrucci et al. (2013)
16	*Proboscia*	Cylinder	$V = \pi/4 * d^2 * h$	Hillebrand et al. (1999); Olenina et al. (2006); Vadrucci et al. (2013)

17	*Rhizosolenia*	Cylinder	$V = \pi/4 * d^2 * h$	Hillebrand et al. (1999); Olenina et al. (2006); Vadrucci et al. (2013)
18	*Skeletonema*	Cylinder	$V = \pi/4 * d^2 * h$	Olenina et al. (2006); Vadrucci et al. (2013)
19	*Thalassiosira*	Cylinder	$V = \pi/4 * d^2 * h$	Hillebrand et al. (1999); Olenina et al. (2006); Vadrucci et al. (2013)
	**Pennate**			
1	*Cylindrotheca*	Prolate spheroid + two cylinder	V = sum of partial volumes	Vadrucci et al. (2013)
2	*Diploneis*	Rotational Ellipsoid	$V = \pi/6 * d^2 * h$	Olenina et al. (2006)
3	*Entomoneis*	Elliptic prism with transapical constriction	$V \approx \pi/4 * a * b * c$; (c = 79% b; from Leblanc estimates)	Sun and Liu (2003)
4	*Fragilaria*	Half parallelepiped	$V = l * w * h/2$; (h = 89% w; from Olenina estimates)	Olenina et al. (2006)
5	*Grammatophora*	Oval cylinder	$V = \pi/4 * d_1 * d_2 * h$; ($d_2$ = 50% d_1; from Olenina estimates)	Olenina et al. (2006)
6	*Gyrosigma*	Prism on parallelogram base	$V = ½ a * b * c$; (c = b; from Leblanc estimates)	Sun and Liu (2003)
7	*Haslea*	Prism on elliptic base	$V = \pi/4 * a * b * c$; (c = 62% b; from Leblanc estimates)	Sun and Liu (2003)
8	*Lioloma*	Cylinder	$V = \pi/4 * d^2 * h$	Hillebrand et al. (1999); Vadrucci et al. (2013)
9	*Mastogloia*	Oval cylinder	$V = \pi/4 * d_1 * d_2 * h$; ($d_2 = d_1$; from Olenina estimates)	Olenina et al. (2006)
10	*Meuniera*	Parallelepiped	$V = l * w * h$; (h = 78% w; from Leblanc estimates)	Olenina et al. (2006)
11	*Navicula*	Prism on elliptic base	$V = \pi/4 * a * b * c$; (c = 76% b; from estimates of this study)	Hillebrand et al. (1999)
12	*Nitzschia*	Prism on elliptic base	$V = \pi/4 * a * b * c$; (c = b; from Olenina estimates)	Vadrucci et al. (2013)

13	*Pinnularia*	Rectangular box	V=a * b * c; (c = b; from Leblanc estimates)	Sun and Liu (2003)
14	*Pleurosigma*	Prism on parallelogram base	V = ½ a * b * c; (c = b; from Leblanc estimates)	Sun and Liu (2003)
15	*Pseudo-nitzschia*	Prism on parallelogram base	V = ½ a * b * c; (c = 50% b; from estimates of this study)	Hillebrand, et al. 1999
16	*Thalassionema*	Rectangular box	V=a * b * c; (c = 65%b; from Leblanc estimates)	Sun and Liu (2003)
17	*Thalassiothrix*	Rectangular box	V=a * b * c; (c = 67%b; from Leblanc estimates)	Sun and Liu (2003)
18	*Tropidoneis*	Prism on elliptic base	V = π/4 * a * b * c; (c = b; from Leblanc estimates)	Sun and Liu (2003)
