# Supplementary figures and images for: Cell carbon content and biomass assessments of dinoflagellates and diatoms in the oceanic ecosystem of the Southern Gulf of Mexico
Source: PLoS One. 2021 Feb 17;16(2):e0247071. doi: 10.1371/journal.pone.0247071 (PMC7888629; doi:10.1371/journal.pone.0247071)

## Dinoflagellates

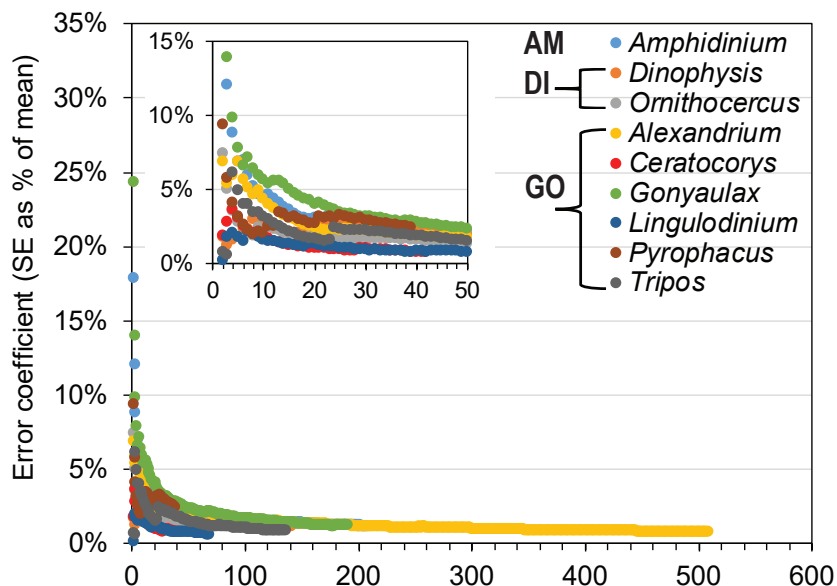

## Diatoms

### Centric

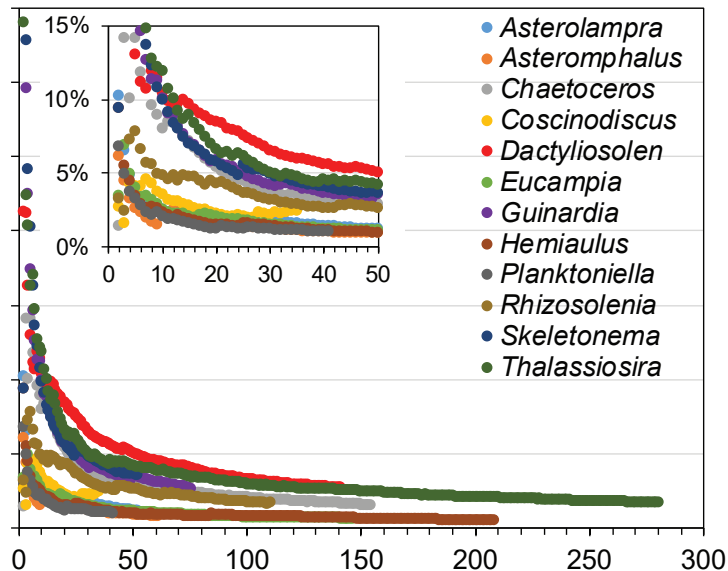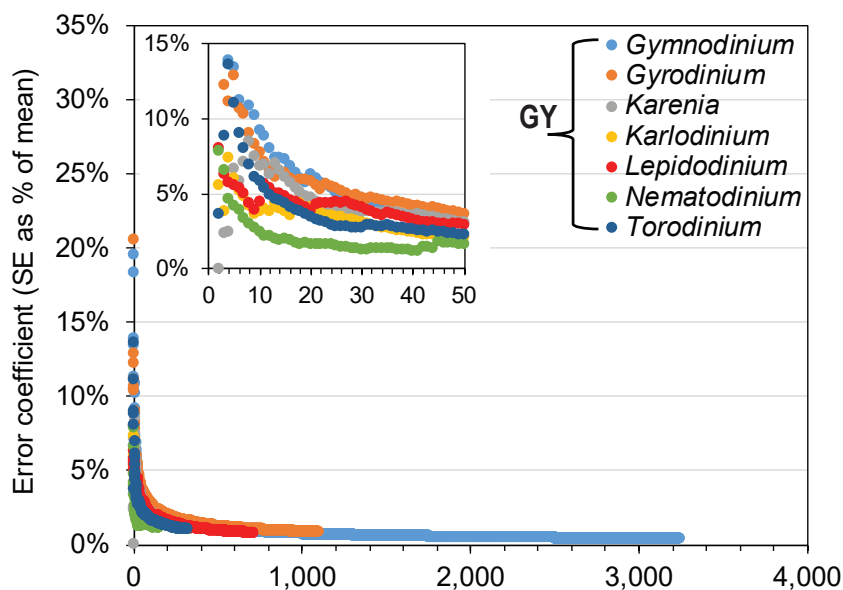

### Pennate

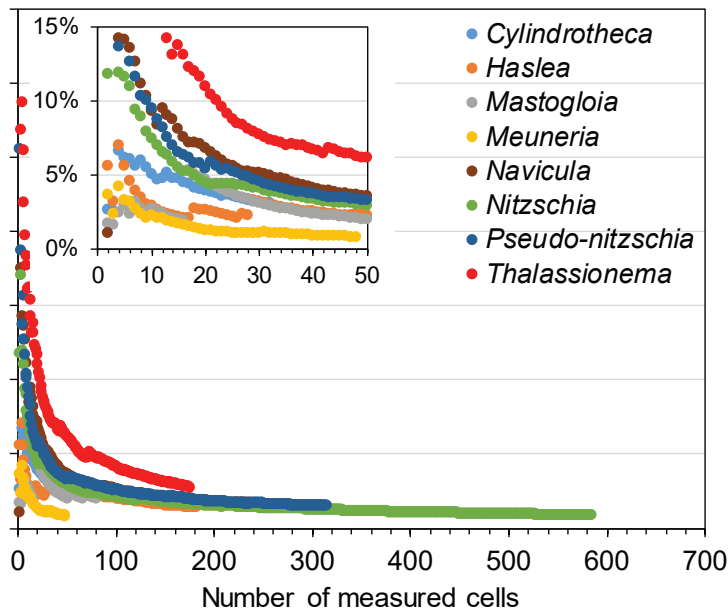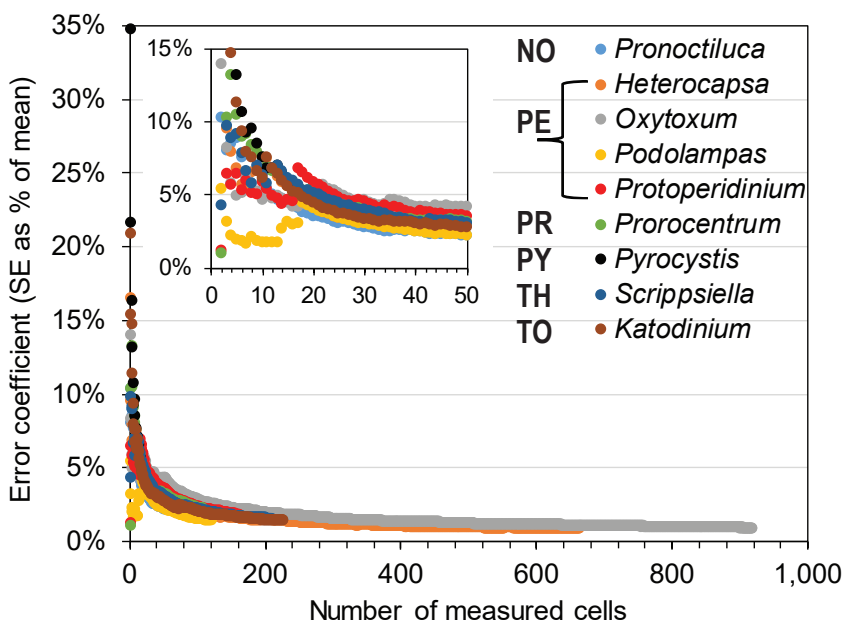

Supplement: S1 Fig — The standard error (SE) expressed as percentage of the mean for genera of dinoflagellates (left panels) and diatoms (right panels) plotted in function of the total number of cells measured per genus. Inset panels show error coefficients from the first 50 cells measured. Dinoflagellate orders: AM, Amphidiniales; DI, Dinophysales; GO, Gonyaulacales; GY, Gymnodiniales; NO, Noctilucales; PE, Peridiniales; PR, Prorocentrales; PY, Pyrocystales; TH, Thoracosphaerales; TO, Tovelliales. (PDF) [file pone.0247071.s002.pdf]

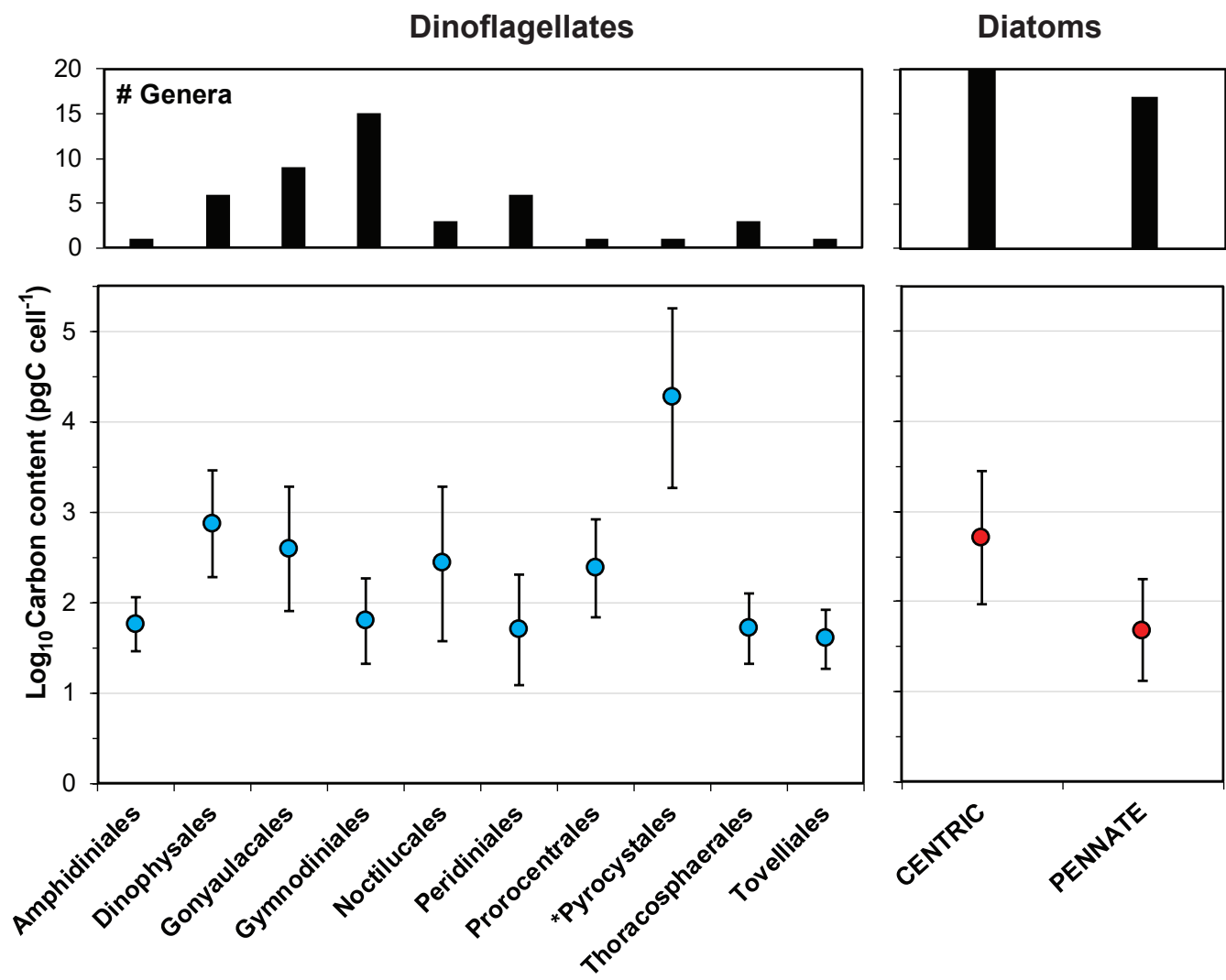

Supplement: S2 Fig — Average log-values of carbon per cell (pg C cell-1; lower panels) for dinoflagellate orders (blue points) and diatom shapes (red points). The total number of genera per dinoflagellate order and diatom shape is indicated at the upper panels (black bars). *Dinoflagellate order with less than 30 individuals. (PDF) [file pone.0247071.s003.pdf]

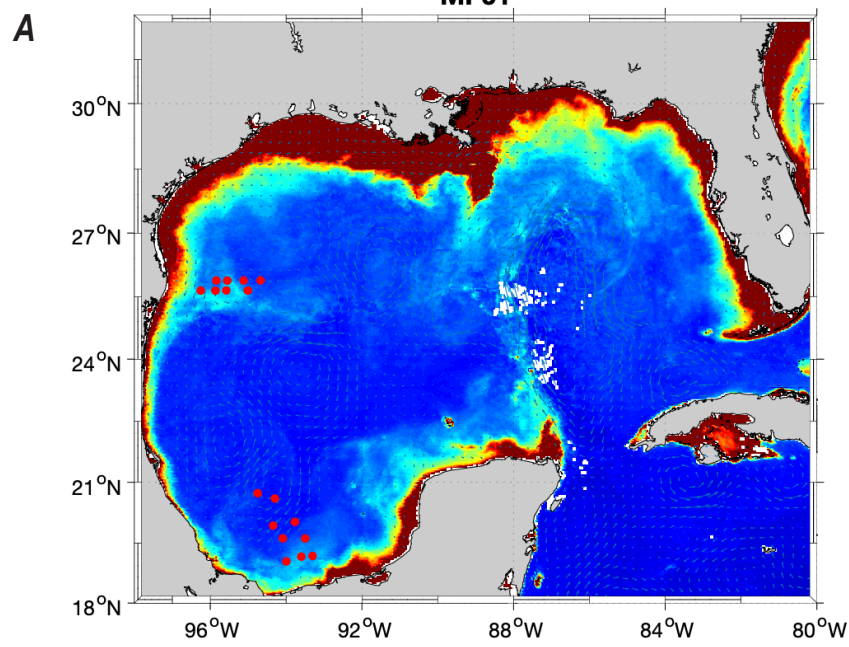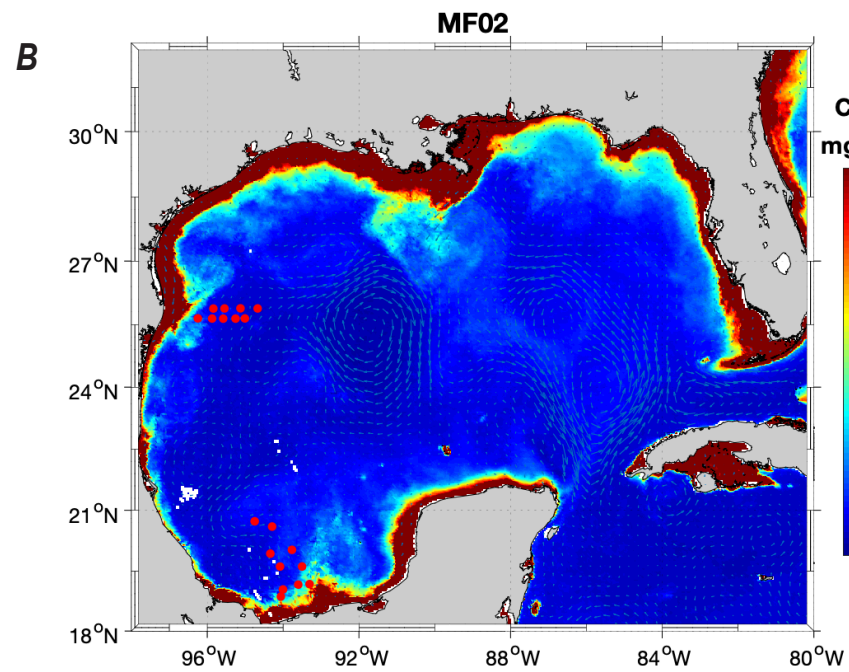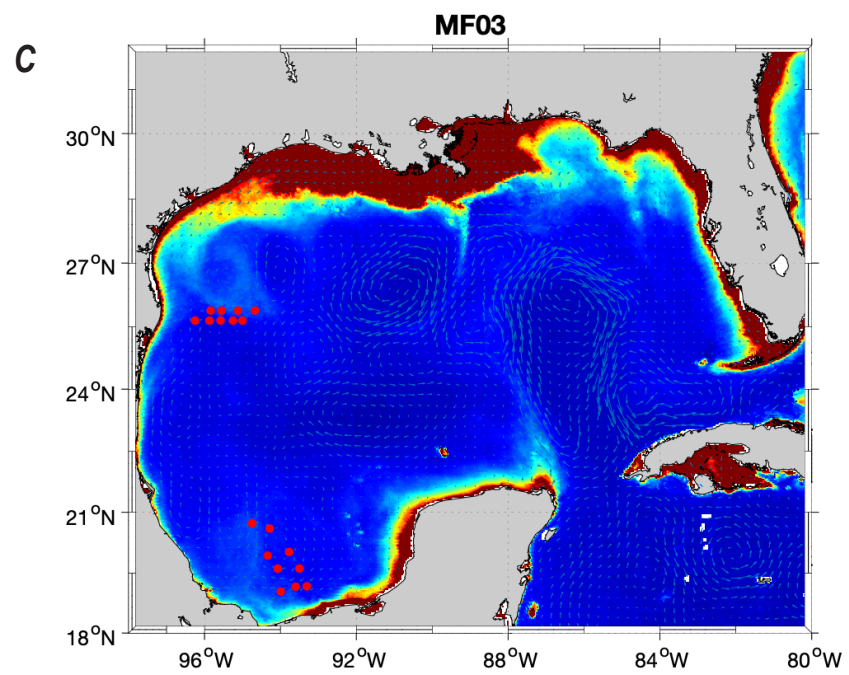

Supplement: S3 Fig — Average images of cruise-mean daily mapped chlorophyll-a concentration (CHL; mg m-3) for (A) late winter 2016 (MF01), (B) late summer 2016 (MF02) and (C) spring 2018 (MF03). Overlaid vectors indicate geostrophic currents as derived from the cruise-mean maps of sea level anomaly (MSLA). Sea color and MSLA images were obtained from the Copernicus Marine Environment Services server (marine.copernicus.eu). For each cruise, sampling stations are indicated as red dots. (PDF) [file pone.0247071.s004.pdf]
